# Sahara Desert sand “Chitligsan”: characterisation and assessment of antibacterial activity and cytotoxicity

**DOI:** 10.1039/d5ra04969e

**Published:** 2026-01-15

**Authors:** Gamze Ertunc, Ebru Yilmaz, Dilan Celebi-Birand, Busra Kilic, Seyma Nigiz, Evren Cubukcu, Ceren Ozkul, Halil Murat Aydin, Memed Duman

**Affiliations:** a Hacettepe University, Institute of Science, Nanotechnology and Nanomedicine Division Ankara Turkey memi@hacettepe.edu.tr; b Molecular Biology Section, Department of Biology, Faculty of Science, Hacettepe University Beytepe Ankara Turkey; c Hacettepe University, Faculty of Pharmacy, Department of Pharmaceutical Microbiology Ankara Turkey; d Hacettepe University, Faculty of Engineering, Department of Geological Engineering Beytepe Campus Ankara Turkey; e Bioengineering Division and Centre for Bioengineering, Institute of Science, Hacettepe University Beytepe Ankara Turkey; f Department of Molecular Biology and Genetics, Bilkent University Ankara Turkey

## Abstract

Antimicrobial resistance presents a significant global health concern, rendering antimicrobial therapies less effective and complicating the management of infectious diseases. To address this challenge, the utilisation of natural alternatives, particularly metal oxide-based nanomaterials, has emerged as a promising strategy due to their antimicrobial activity and favourable physicochemical properties, without inducing antimicrobial resistance. In this study, Chitligsan, a naturally occurring hybrid compound extracted from the sand of the Sahara Desert, was characterised and evaluated in terms of its antimicrobial activity and biocompatibility. The elemental and mineral composition of Chitligsan was investigated using FTIR, SEM-EDS, XRF, XRD, and Py-GC-MS analyses. The results indicated the presence of diverse metal oxide compounds, including Fe_2_O_3_, CaO, and SiO_2_. Additionally, the antimicrobial efficacy of Chitligsan against several pathogenic bacteria, namely *Pseudomonas aeruginosa*, *Escherichia coli*, and *Staphylococcus aureus*, was evaluated. The cytotoxic potential of Chitligsan was assessed in the L929 fibroblast cell line using a scratch assay. Chitligsan exhibited antimicrobial activity against all tested bacterial species, as demonstrated by growth inhibition in the agar well diffusion assay. Furthermore, Chitligsan showed a high level of cytocompatibility, with cell viability exceeding 90%, as confirmed by the MTT assay. In conclusion, owing to its unique hybrid composition, low-cost availability, and dual antibacterial–biocompatible profile, Chitligsan may offer a novel platform for the development of sustainable antimicrobial systems.

## Abstract 2

Chitligsan is a Sahara-derived biopolymer nanomaterial enriched with metal oxides. Analytical characterization confirmed its composition, while assays showed low cytotoxicity, scratch closure and antibacterial activity.

## Graphical abstract

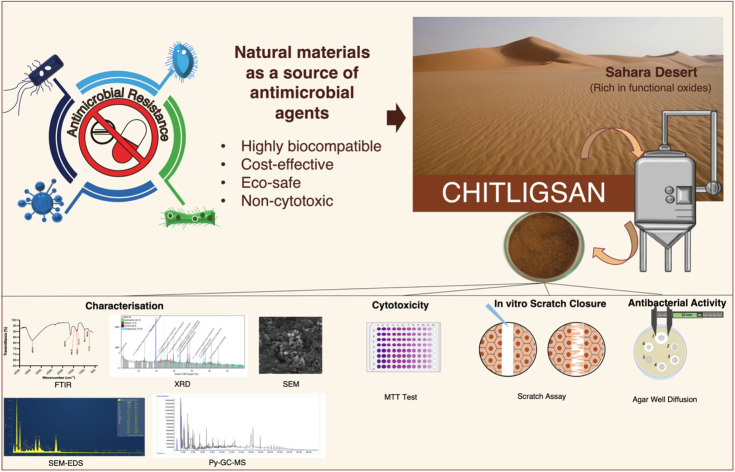

## 1 Introduction

The rise of antimicrobial-resistant bacterial infections has emerged as one of the most severe threats to global public health.^1^ It is estimated that by 2050, antimicrobial resistance (AMR), ‘a silent pandemic’, will cause approximately 50 million deaths globally and impose a cost of 100 trillion dollars on the global economy.^2,3^ In recent years, infection management has become more challenging due to the extensive and rapid spread of resistance mechanisms against traditional antibiotics. To address this growing challenge, the use of natural alternative therapeutic strategies has emerged as a prominent area of research. Among these, the integration of nanotechnology into antibacterial therapies, particularly through metal oxide-based nanomaterials, holds promise owing to their broad-spectrum antimicrobial activity and diverse physicochemical features.^4^ In addition to their antibacterial potential, metal oxide nanoparticles (MONPs) have attracted interest due to their reactivity, extensive surface area, and small size. The antibacterial properties of metal oxides—such as ZnO, MgO, TiO_2_, and Fe_2_O_3_—have been attributed to several mechanisms, including the disruption of bacterial membranes, interference with metabolic pathways, disturbance of redox equilibrium *via* ion release, and induction of oxidative stress by the generation of reactive oxygen species (ROS).^5,6^ Although MONPs are distinguished as potent antimicrobial agents, they have some limitations regarding biocompatibility and environmental safety. MONPs offer versatility, since they can be sourced naturally or synthesised. While synthetic production provides control and consistency, naturally derived MONPs are more environmentally friendly and cost-effective. The decomposition of these nanoparticles in biological environments and the subsequent release of ions may result in the generation of reactive oxygen species (ROS), leading to oxidative stress.^7^ Consequently, the pursuit of safer and more sustainable nanomaterials has become the central focus in numerous studies, particularly those exploring nanomaterials derived from natural or environmentally neutral mineral sources.^8–11^

Nanomaterials derived from environmentally sourced materials, such as clays and other mineral-rich substances like bentonite and zeolites, have been proposed as lower-risk and more biocompatible alternatives, mainly due to their natural origin, reduced toxicity and overall environmental impact. Interestingly, historical practices across different ancient civilisations, including the Egyptians and Mesopotamians who employed iron and calcium oxide-rich clays for their antimicrobial properties, align with contemporary research validating the infection-preventive and wound-healing attributes of clay minerals and various soil compositions.^12^ This continuity highlights the long-standing therapeutic relevance of naturally occurring mineral-based materials. Indeed, recent studies on the French green clay, which contains an iron-rich mineral, have suggested its antimicrobial activity against *Pseudomonas aeruginosa* and *Mycobacterium marinum*, as well as against penicillin-resistant *Staphylococcus aureus*.^13^ The aforementioned findings suggest that these naturally sourced materials may offer cost-effective and sustainable alternatives for treating infections. In nature, mineral–organic interactions in iron-rich sands can lead to ligand-assisted Fe(iii)/Fe(ii) redox cycling, promoted by moisture, sunlight, and fungal-derived organic acids.^14^ In light of these biogeochemical phenomena, the synthesis of Chitligsan was designed to produce a mineral composite containing biopolymer–metal interactions. This composite exhibits redox activity similar to that observed in natural iron-rich sands.^14^

Chitligsan, a naturally occurring hybrid compound extracting in the sands of the Sahara Desert, represents one such promising biocompatible material. Although current data on Chitligsan remains limited, initial observations indicate a potential for supporting wound healing^15^ and exhibiting cellular compatibility. The biological effect and its biocompatibility are believed to originate from the accumulation of crustaceans, diatoms, and other aquatic organisms in the region.^15^ Given its complex composition and potential biological ramifications, further comprehensive analysis is required to fully elucidate the material's capabilities.

Assessing naturally produced nanomaterials only for their antibacterial efficacy may be insufficient for biological applications. Therefore, a comprehensive safety evaluation is required through systematic examination of their biocompatibility at the cellular level. To implement these nanomaterials in biomedical and environmental applications, and ensure their functionality and reliability, comprehensive characterisation of their physicochemical properties, as well as their composition, is also critical. In this context, X-ray diffraction (XRD) is extensively used for determining a material's crystalline structure, crystallinity level, and phase composition. Furthermore, surface characteristics, including particle morphology, size distribution, and elemental composition, may be elucidated using the integration of scanning electron microscopy (SEM) and energy-dispersive X-ray spectroscopy (EDS). X-ray fluorescence (XRF) enables semi-quantitative analysis of heavy metal concentrations, ranging from fluorine (F) to uranium (U), in both percentage (%) and parts per million (ppm). The Fourier-transform infrared spectroscopy (FTIR) method, on the other hand, analyses the functional groups and chemical bond structure on the surface, providing important information about the molecular-level properties of the materials.^16,17^ Pyrolysis-gas chromatography-mass spectrometry (Py-GC-MS) was further employed to identify volatile organic compounds generated during thermal decomposition, offering insight into the polymeric and carbonaceous constituents of the material. Together, these characterisation techniques facilitate the elucidation of potential interactions between nanostructured materials and biological systems, while also revealing their toxicological profiles.

In the present study, Chitligsan was extensively characterised using the previously mentioned analytical techniques—XRD, SEM-EDS, XRF, Py-GC-MS, and FTIR—to examine its structural and chemical properties in detail. In addition to physicochemical examination, the antibacterial activity of Chitligsan against pathogenic bacteria (*Escherichia coli* ATCC 25922, *Pseudomonas aeruginosa* ATCC 27853, and *Staphylococcus aureus* ATCC 29213), alongside its cytotoxicity and impact on cell viability, was evaluated to gain further insight into its compatibility with biological systems. Ultimately, this study aimed to assess the biological compatibility and reliability of Chitligsan through a thorough analysis of its structural, chemical, and biological attributes.

## 2 Experimental

### 2.1. Reagents

Dimethyl sulfoxide (DMSO), citric acid (≥98%), [3-(4,5-dimethylthiazol-2-yl)-2,5-diphenyltetrazolium bromide] (MTT), Dulbecco's Modified Eagle Medium (DMEM), glutaraldehyde solution (25%), chitosan, Phosphate Buffered Saline (PBS) and ethanol (HPLC grade) were purchased from Sigma-Aldrich (Missouri, US). Foetal Bovine Serum (FBS) and Mueller–Hinton agar (MHA) were purchased from Thermo Fisher (Massachusetts, US). Deionised water used throughout the experiments was purified with the aquaMAX™-Basic 360 Series water system (Young Lin Instrument, South Korea).

### 2.2. Preparation of Chitligsan

Chitligsan, a proprietary chitosan-based composite material provided by Chitlig Energy Generation and Marketing Co. Inc. (Ankara, Türkiye), was used in this study. Chitligsan was prepared using the method described in European patent (Patent No: EP3976662A1). Briefly, it is extracted by mixing Sahara-origin mineral powder with oxalic acid produced by *Aspergillus niger* strains in a reactor, using a 10 (solid)/1 (liquid) ratio with distilled water, while maintaining the mixture at pH 2. After separating the liquid phase from the solid material, the solution is transferred to a second reactor, where sodium hydroxide is added in a controlled manner to agglomerate the nanostructures in the solution. Through this two-stage process, a high-purity Chitligsan suspension is obtained. Later, a 50 mL of this Chitligsan stock solution (100 mg mL^−1^) was centrifuged at 2000 rpm to separate the solid and liquid phases. The solid phase was subsequently transferred to a Petri dish and dried in an oven at 80 °C for 2 h. The resulting Chitligsan powder was then utilised for characterisation studies and subsequent biological analyses.

### 2.3. Characterisation of Chitligsan

#### 2.3.1. Scanning electron microscopy (SEM)

Morphological properties of Chitligsan were analysed using a Gaia 3 (Tescan, Czech Republic) SEM with a field emission electron gun. Prior to imaging, carbon sputter-coating (5 nm) was applied to the samples. For elemental analysis, the SEM was equipped with an XMax 150 (Oxford Instruments, UK) Energy Dispersive X-ray Spectroscopy (EDS) detector (SEM-EDS). The instrument was operated under 10 kV accelerating voltage, 1–3 nA beam current at 9 mm working distance.

#### 2.3.2. Dynamic light scattering (DLS) and zeta potential

Dried Chitligsan samples were dispersed in 0.1 M citric acid solutions adjusted to different pH values (2.3, 6, 9, and 12). Dynamic light scattering (DLS) and zeta potential measurements were performed at room temperature using a Zetasizer Nano ZSP (Malvern Instruments, UK) equipped with a folded capillary cell. Prior to analysis, all samples were filtered through a 0.45 µm membrane filter, allowed to equilibrate for 5–10 min, and then carefully loaded into the cell to avoid bubble formation.

#### 2.3.3. X-ray diffraction (XRD) and X-ray fluorescence spectroscopy (XRF)

The Chitligsan samples were subjected to XRD (Aeris, Malvern Panalytical, Netherlands) analysis to determine the elemental composition and relative abundances. The diffraction patterns were recorded with a selected range of 0° (2*θ*) to 70° (2*θ*) scanning search parameter, and with a step size of 0.1° min^−1^ using Cu-Kα radiation (*λ* = 1.5406 Å, 45 kV, 40 mA). Identification of crystalline phases were performed using HighScore platform with pre-installed crystal database. Complementary elemental composition was analysed *via* XRF spectroscopy (Epsilon 4, Malvern Panalytical, Netherlands).

#### 2.3.4. Fourier transform infrared spectroscopy (FTIR)

FTIR analysis was performed to identify functional groups and bond types and evaluate the chemical structure of Chitligsan. The FTIR spectrum of the sample was recorded using a Nicolet™iS50 (Thermo Scientific™, US) spectrometer within the range of 500–4000 cm^−1^.

#### 2.3.5. Pyrolysis-gas chromatography-mass spectrometry (Py-GC-MS)

For the analysis, approximately 10 mg of dry sample was placed in a 100 mL stainless-steel flask under vacuum and heated at 600 K for 1 h in an oil bath. After cooling to room temperature, volatile compounds were extracted with CH_2_Cl_2_ (3 × 10 mL), dried over anhydrous MgSO_4_, filtered, and concentrated to ∼0.5 mL under a gentle nitrogen stream. GC-MS measurements were performed using an Agilent 7890 gas chromatograph coupled with a 5977C mass spectrometer (Agilent Technologies, USA) equipped with a DB-1 fused silica capillary column (30 m × 0.25 mm, 0.25 µm film thickness). The column temperature was programmed from room temperature to 543 K at a rate of 3 K min^−1^. Helium was used as the carrier gas at a flow rate of 1.0 mL min^−1^, with the injector temperature maintained at 600 K. Mass spectra were acquired under electron ionization (70 eV) in the *m*/*z* range of 33–500. Compound identification was carried out by comparison with the Wiley mass spectral library.

### 2.4. Evaluation of the antimicrobial activity

Antimicrobial activity of Chitligsan was evaluated using agar well diffusion assay against *Escherichia coli* ATCC 25922, *Pseudomonas aeruginosa* ATCC 27853, and *Staphylococcus aureus* ATCC 29213. Bacteria were incubated on blood agar overnight at 37 °C, and a standard bacterial suspension was prepared by adjusting the turbidity of the suspension to match the 0.5 McFarland standard (∼1.5 × 10^8^ CFU mL^−1^). For the experiments, 1 mg of dried Chitligsan was dissolved in 1 mL distilled water (referred to as the Chitligsan-DI extract) or 1 mL 0.1 M citric acid (referred to as the Chitligsan-acidic extract). Ciprofloxacin (CIP; 5 µg mL^−1^) was used as a positive control, while 0.1 M citric acid and distilled water served as negative controls. Muller–Hinton Agar (MHA) plates were uniformly inoculated with the bacterial suspensions. Subsequently, a 6 mm hole was punched aseptically with a sterile cork borer in the agar, and wells were filled with 50 µL of each test sample or control. The plates were incubated at 37 °C for 24 hours. The zones of inhibition were measured in millimetres to assess antimicrobial efficacy.

Besides the agar diffusion assay, SEM imaging was employed to observe morphological changes in bacterial cells following exposure to Chitligsan. Prior to SEM analysis, bacterial cells were fixed to preserve cellular morphology and prevent structural collapse during sample preparation. The test group was treated with 1 mg per mL Chitligsan, prepared in 0.1 M citric acid, and mixed with the bacterial suspension. A second group was exposed to 0.1 M citric acid solution to assess solvent-related effects, while an untreated bacterial culture served as the control group. For surface adhesion studies, 1 mL of the bacterial–Chitligsan mixture was dispensed onto sterile glass slides and incubated for 4 h at room temperature to allow cell attachment. The slides were then gently rinsed three times with PBS to remove non-adherent material. Adherent cells were subsequently fixed in 2.5% (v/v) glutaraldehyde prepared in 0.1 M phosphate buffer (pH 7.2–7.4) for 30 min at room temperature. Then, the samples were dehydrated through a graded ethanol series (30%, 50%, 70%, 90%, and 100%; 30 min each). After the final dehydration step, samples were air-dried at room temperature and examined by scanning electron microscopy (Gaia 3, Tescan, Czech Republic).

### 2.5. *In vitro* cytocompatibility: MTT assay

MTT [3-(4,5-dimethylthiazol-2-yl)-2,5-diphenyltetrazolium bromide] assay was carried out as previously reported.^18^ L929 mouse fibroblast cells were maintained in RPMI-1640 and supplemented with 10% FBS and 1% Pen per Strep solution. Cells (1 × 10^4^ cells per well) were seeded in 96-well plates and treated with decreasing concentrations (50 mg mL^−1^,25 mg mL^−1^,12.5 mg mL^−1^, 6.25 mg mL^−1^) of Chitligsan stock solution (100 mg mL^−1^) for 2 h. As a positive control, 2.5% DMSO was used. Following incubation, MTT (0.5 mg mL^−1^) was added to the medium and incubated for 3 h at 37 °C. To dissolve the formazan crystals, DMSO was added to the wells. The optical density of formazan crystals was measured at 570 nm using a microplate reader (Biotek, US).

### 2.6. Scratch assay

To assess cell migration in an *in vitro* scratch assay following Chitligsan treatment, L929 cells (2 × 10^4^ cells per well) were seeded in 96-well plates. The cell monolayer was scratched horizontally with a sterile 200 µL pipette tip. The cell monolayer was then washed with 1× PBS to remove debris. The Chitligsan solution (100 mg mL^−1^) was added to the plates, and their effects on cell migration were assessed. The plates were incubated at 37 °C with 5% CO_2_. Coordinated images were captured at three different time points (0 h, 24 h and 48 h) using an inverted microscope (Leica, DM IL LED, Germany). The percentage of scratch closure was calculated using ImageJ (NIH) software according to the formula (*T*_0_ − *T*_*x*_)/*T*_0_ × 100, where *T*_0_ represents the initial scratch area and *T*_*x*_ represents the scratch area at each time point.

### 2.7. Statistical analysis

Statistical analysis was performed using Prism 8 (GraphPad Software Inc., California, US), with a significance threshold set at *p* < 0.05. A one-way ANOVA with Bonferroni's multiple comparison test was performed for the across-group comparison. The results are presented as the mean ± standard error from three independent biological replicates. Significance levels were as follow: *p* < 0.05 (*); *p* < 0.01 (**); *p* < 0.001 (***); and *p* < 0.0001 (****).

## 3 Results and discussion

### 3.1. Characterisation of Chitligsan

The characterisation experiments were initiated by examining surface morphology. This analysis was conducted on dried Chitligsan.

**Fig. 1:**
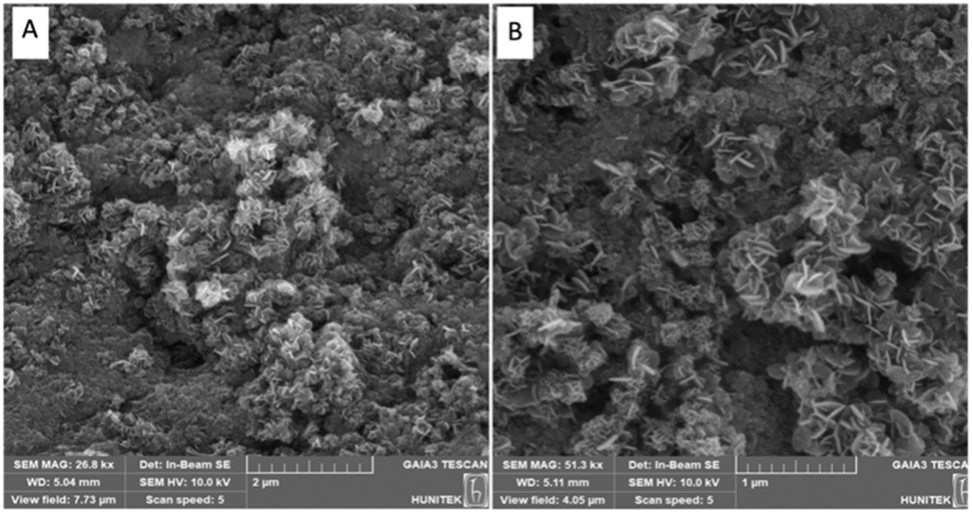
SEM images of the mineral–biopolymer composite showing nanoflower-like surface morphology. (A) Low-magnification SEM image illustrating the overall heterogeneous and hierarchically organized structure with widespread nanoflower-like formations. (B) Higher-magnification (zoom-in) SEM image of the highlighted region in (A), revealing the detailed flower-like structure composed of radially arranged petal-like nanosheets.

Scanning electron microscopy (SEM) analysis revealed that the mineral–biopolymer composite exhibits a heterogeneous and hierarchically organized surface morphology (Fig. 1A). The surface is dominated by irregular agglomerates within which well-defined nanoflower-like structures are widely distributed. These nanoflower-like architectures consist of radially oriented, petal-like subunits assembled around a central core, forming three-dimensional flower-shaped morphologies. Moreover, these nanoflower domains provide significantly increased accessible surface area, enhanced structural complexity, and high exposure of functional groups.

A higher-magnification SEM image (Fig. 1B), representing a zoomed-in view of the selected region in Fig. 1A, provides a clearer visualization of these structures. Individual nanoflowers display an average overall diameter in the range of approximately 200–400 nm, while the constituent petal-like nanosheets exhibit estimated thicknesses of 10–30 nm. The close packing and interconnection of these nanosheets contribute to the formation of a porous and rough surface topology.

The formation of nanoflower-like morphologies is attributed to the synergistic interaction between the mineral phase and the biopolymer matrix.^19^ Functional groups present along the biopolymer chains (*e.g.*, hydroxyl, amino, or carboxyl groups) likely act as nucleation and growth-directing sites for mineral deposition, promoting anisotropic crystal growth and self-assembly into flower-like hierarchical structures^.^^19,20^ This biomineralization-driven organization results in a significantly increased specific surface area and enhanced surface roughness.^20^

Such nanoflower-like architectures have been frequently reported in biopolymer-assisted mineralization systems and are known to provide advantageous micro/nano-environments for biological interactions, including improved cell adhesion, enhanced bioactivity, and controlled ion or molecule release.^20^ Taken together, the observed SEM morphology suggests an effective integration of the mineral and biopolymer phases, consistent with previously reported biopolymer-assisted biomineralization systems.

The colloidal behaviour and surface charge characteristics of Chitligsan were evaluated using hydrodynamic diameter of particles and zeta potential measurements, as summarized in Table 1 and detailed in the SI (Fig. S1–S4). The zeta potential of Chitligsan showed a strong dependence on pH. It remained nearly neutral under acidic conditions (pH 2.3–6), reached its lowest negative value at pH 9, and became positive at pH 12. This reversal in surface charge reflects pH-driven ionization changes due to the protonation and deprotonation of functional groups. At near-neutral pH (6–9), the zeta potential approached zero and the particle's hydrodynamic diameter increased noticeably, indicating reduced charge stabilization and a higher tendency to aggregate. Limited colloidal stability is a typical characteristic of oxide-based hybrid systems, particularly when the zeta potential remains below 30 mV.^21^ Our zeta potential values are not only below 30 mV but remain within *ζ* < 10 mV, indicating near-neutral surface charge and very weak electrostatic stabilization, which is consistent with the observed aggregation behavior. Under such conditions, electrostatic repulsion is weak, and multivalent cations such as Ca^2+^, Mg^2+^, and Fe^3+^ in the matrix or suspension medium can promote particle aggregation by partially neutralizing surface charges.^22,23^ DLS measurements nevertheless showed a dominant structure population (72–94% intensity) throughout the tested pH range, indicating that the system did not fully precipitate but instead underwent reversible aggregation. The partial stability observed is likely related to the protonated amino groups of chitosan, which impart polyelectrolyte behavior and contribute to electrostatic repulsion under acidic to mildly acidic conditions.^24^ Previous reports also reported that surface modification with citric acid or nonionic surfactants can further improve dispersion stability through electrostatic or steric repulsion.^25^

**Table 1:** Effect of pH on the particle size distribution, PDI, and surface charge (zeta potential) of Chitligsan

pH	Hydrodynamic diameter (nm)	Intensity (%)	PDI	Zeta potential (mv)
2.3	368.7	94.3	0.835	−0.409
6	362.3	81.2	0.594	−1.74
9	645.4	71.9	0.620	−6.29
12	1007	94.4	0.535	4.33

Our DLS data supported these observations, providing further insight into pH-dependent aggregation behaviour. The hydrodynamic diameter of Chitligsan particles increased noticeably with increasing pH, from about 370 nm under acidic and neutral conditions (pH 2.3–7) to roughly 645 nm at pH 9 and over 1000 nm at pH 12. This pattern reflects a pH-dependent aggregation tendency, as particle growth became more pronounced under neutral to alkaline conditions. The consistently high PDI values (0.5–0.8) indicate a broad size distribution and partial heterogeneity within the dispersion.

**Fig. 2:**
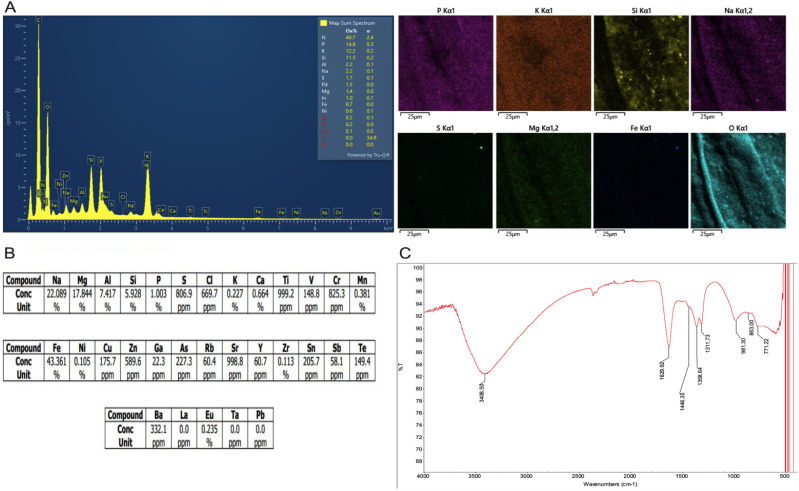
Characterisation of elemental composition and spectra of Chitligsan. (A) Constituent elements and EDS image patterns, (B) XRF results, and (C) FTIR spectra of Chitligsan.

Next, the elemental composition of the Chitligsan was analysed by utilising SEM-EDS. The elemental distribution and corresponding EDS spectra are provided in Fig. 2A. According to the EDS analysis, the major element group includes nitrogen (N; 49.7%), phosphorus (P; 14.8%), potassium (K; 12.2%), silicon (Si; 11.5%), aluminium (Al; 2.2%), sodium (Na; 2.2%), sulfur (S; 1.7%), magnesium (Mg; 1.4%), and iron (Fe; 0.7%), while the minor element group comprises zinc (Zn; 0.2%), titanium (Ti; 0.2%) and calcium (Ca; 0.1%).

Several studies have reported the notable abundance of inorganic compounds such as iron(iii) oxide (Fe_2_O_3_), magnesium oxide (MgO), calcium oxide (CaO) and silicon dioxide (SiO_2_) in the Sahara Desert.^26,27^ In mineral-rich sands and soils, these oxides have been shown to play a significant role in ROS production.^28^ Consistent with these findings, our results suggested that Chitligsan also contain significant amounts of silicon oxide and iron oxide compounds. To validate this hypothesis and acquire a more detailed elemental profile of the material, XRF analysis was conducted on the dried Chitligsan sample to identify the constituent elements and their quantitative ratios (Fig. 2B). The results showed that iron was the dominant element, making up 43.36% of the total content. However, due to the limitations of employed techniques (EDS and XRF), the relative concentrations of ferric and ferrous iron could not be measured but expressed as total Fe. The sample also contained notable amounts of sodium (20%), magnesium (17.84%), aluminium (7.42%), and silicon (5.93%). In addition, trace levels of heavy metals such as chromium, barium, copper, and strontium were detected. Collectively, these results corroborated the presence of diverse metal oxide compounds in Chitligsan.

The metallic elements (*e.g.*, Na, Ca, Fe) identified through XRF are commonly present in nature in crystalline phases and often in hydrated forms. There is a tendency for these metal ions to attach themselves to the hydroxyl groups on the biopolymer surface, which results in observable shifts or intensity changes in the O–H stretching region in the FTIR spectrum.^29^ To elucidate these interactions and identify the characteristic functional groups associated with metal-oxide bonding in Chitligsan samples, FTIR spectroscopy was employed (Fig. 2C). The resulting spectra confirmed the presence of diverse metal oxide compounds in Chitligsan. In addition to a prominent absorption band at 3408.50 cm^−1^, corresponding to the stretching vibrations of hydroxyl groups (OH), a less intense band observed at 1629.92 cm^−1^ was interpreted as the bending of H–O–H groups.^30^ This bending mode is typically associated with molecular water adsorbed on the surface or confined within the structure of hydrophilic minerals or metal hydroxides.^29,31^ Thus, the OH bands, spectrum at 3408.50 cm^−1^ and 1629.92 cm^−1^, likely arise from mineral hydration or metal–hydroxyl interactions.^32^ These findings suggested a tendency for sodium, calcium and iron ions to interact with hydroxyl groups, resulting in a decrease in their vibrational frequencies. Moreover, the absorption band at 1443.35 cm^−1^ corresponds to the characteristic asymmetric stretching of carbonate ions (CO_3_^2−^), while the bands at 1358.64 cm^−1^ and 1311.73 cm^−1^ are interpreted as indicative of calcite.^33^

Similarly, the presence of phosphorus (P) element in Chitligsan as revealed by the XRF data suggests the presence of phosphate (PO_4_) groups in the structure.^34^ Attributing the phosphate peak at 981 cm^−1^ to a calcium phosphate (Ca_3_(PO4)_2_) compound, potentially β-tricalcium phosphate (β-TCP) (Ca_3_(PO_4_)_2_) or hydroxyapatite (Ca_10_(PO_4_)_6_(OH)_2_), it is plausible that divalent cations such as magnesium (Mg^2+^) or strontium (Sr^2+^) could substitute for calcium (Ca^2+^) within the crystal lattice.^35^ Consequently, this structural configuration may impact the FTIR absorption frequencies. The band observed at 863 cm^−1^ in the infrared spectrum of the sand sample is likely due to symmetric and asymmetric stretching vibrations of Si–O bonds, whereas the absorption peak around 771 cm^−1^ is characteristic of their bending vibrations, and indicates the presence of pure silica (SiO_2_) in the sand sample.

**Fig. 3:**
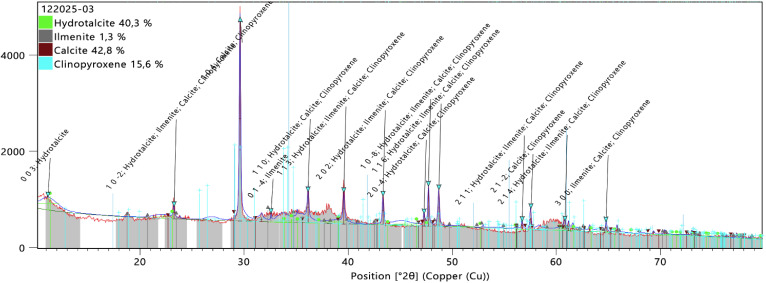
Analysis of the XRD patterns of Chitligsan. Calcite was the most abundant mineral (42.8%), followed by hydrotalcite (40.3%), clinopyroxene 15.6%, and ilmenite 1.3%.

To further elucidate the phase composition and crystalline properties of Chitligsan, XRD analysis was conducted. The identified components and their corresponding percentages are depicted in Fig. 3. The analysis revealed that Chitligsan is mainly composed of calcite (42.8%) and hydrotalcite (40.3%), with minor amounts of clinopyroxene (15.6%) and ilmenite (1.3%).

Calcite (CaCO_3_) is among the most abundant minerals, accounting for nearly 4% of the Earth's crust. Reactions occurring on calcite surfaces are central to many natural geochemical and environmental processes. In addition, calcite is widely utilized in industry, including paper and cement manufacturing, subsurface storage of CO_2_ and nuclear waste, and various stages of oil and gas extraction.^36^ CaCO_3_ is a biocompatible mineral widely used in biomedical applications, including bone graft substitutes and tissue-friendly fillers.^37^ In particular, the calcite polymorph is well established as a biocompatible material and is frequently incorporated into bone-related scaffolds, surface coatings, and drug delivery systems, as it interacts safely with biological tissues.^38^

Hydrotalcite (Mg_6_Al_2_(OH)_16_CO_3_·4H_2_O) was the second major phase identified in Chitligsan, accounting for 40.3% of the composition. This mineral has a layered crystal structure consisting of positively charged hydroxide layers and interlayers composed of carbonate anions and water molecules.^39^ Structurally derived from brucite (Mg(OH)_2_), hydrotalcite mineral incorporates divalent cations Mg^2+^, Fe^2+^, Mn^2+^, Zn^2+^, Cu^2+^, and Ni^2+^ within its structure. It is generally found in geological deposits and forms.^35^ Furthermore, the presence of brucite (Mg(OH)_2_) is significant because a brucite-like layered structure imparts various physicochemical and biological properties. These properties affect the environmental, biomedical or industrial applications of the material, playing a critical role in its surface interactions, ion exchange and reactivity.^40–42^

The third most common phase, Clinopyroxene accounted for 15.6% of the crystalline composition of Chitligsan. Clinopyroxene is a Ca–Mg–Fe silicate widely recognized as a rock-forming mineral in mantle and crustal rocks. It accommodates significant amounts of Na, Ca, Cr and Ti in its structure and plays an important role in element partitioning during melting and crystallization processes.^43,44^

The last crystalline phase identified in Chitligsan was ilmenite (FeTiO_3_). Ilmenite is an Fe–Ti oxide that typically occurs as a minor accessory phase in mixed silicate systems. Although mainly described in geological contexts, ilmenite has also been investigated in several applied research areas. For example, alginate films incorporating ilmenite-grafted graphene oxide ilmenite (1%). A exhibited antimicrobial activity and reduced fruit spoilage under visible light.^45^ In another study, ilmenite-derived Fe–O/Ti–O composites have been evaluated as antibacterial agents and were reported to inhibit both Gram-positive (*B. cereus*) and Gram-negative (*E. coli*) bacteria, with stronger effects observed against the Gram-positive strain.^46^

Collectively, the characterization analyses indicate that Chitligsan is a heterogeneous mineral composite dominated by carbonate, layered double hydroxide, and silicate phases, with iron–titanium-containing components present at minor levels. In addition, the detection of iron (Fe) and titanium (Ti) elements by XRF analysis suggests that these components may contribute to the antimicrobial response of Chitligsan. Iron titanium dioxide nanoparticles have been shown to exhibit antimicrobial activity against microorganisms such as *E. coli*, *P. aeruginosa*, and *S. aureus*.^47^ Therefore, subsequent analyses were conducted to assess the antimicrobial efficacy of Chitligsan.

**Fig. 4:**
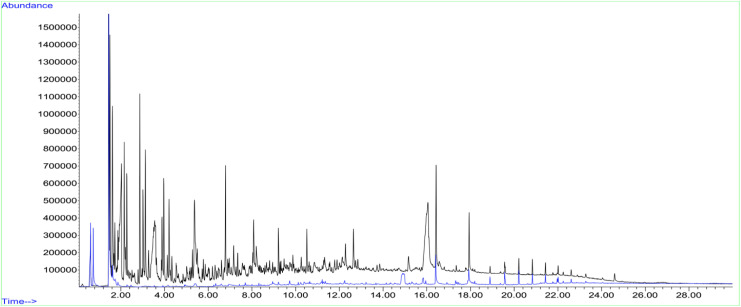
Gas chromatography of volatile compounds generated from pyrolysis of chitosan standard (black trace) and Chitligsan (blue trace).

Py-GC-MS analysis was conducted to compare the thermal breakdown products of the Chitligsan composite with those of reference chitosan (Fig. 4 and SI of Py-GC-MS Spectrum). Due to its abundant amino-sugar backbone, pure chitosan produces a wide variety of volatile fragmentation products when pyrolyzed, leading to a richer peak profile in Py-GC-MS (*e.g.*, pyrazines, pyrroles, and furans).^48^ In contrast, samples with substantial inorganic or metal-oxide content tend to yield fewer volatiles, weakening overall signal intensity.^49^ The black trace in Fig. 4 represents the chromatogram of pure chitosan, whereas the blue trace corresponds to the Chitligsan sample. As shown in the Fig. 4, several overlapping volatile compounds were detected between the two samples, supporting the compositional similarity of chitosan-derived constituents within the composite. Highly diagnostic nitrogen-amide markers such as acetamide or acetamidofuran were not detected at significant intensity. However, the Chitligsan spectrum revealed several nitrogen-containing heterocycles (pyrrole-, pyridine-, and pyrazine-type fragments) together with oxygenated carbohydrate derivatives such as furan, furanone, and oxolanone species. This compositional pattern is consistent with prior reports describing the pyrolytic fragmentation of chitosan, in which pyrazines and pyrroles originate from condensation and cyclization of glucosamine-derived fragments, while furanic compounds result from ring-opening and dehydration of the polysaccharide backbone.^48,50^ The presence of these characteristic pyrolysis products suggests that Chitligsan undergoes similar nitrogen-containing degradation pathways to those of chitosan.^37^ Although these oxygenated compounds are not specific to chitosan, their co-occurrence with nitrogen-containing fragments is in line with the characteristic degradation profile of chitosan. Together, these features support the presence of chitosan-related structural components in the Chitligsan composite.

**Fig. 5:**
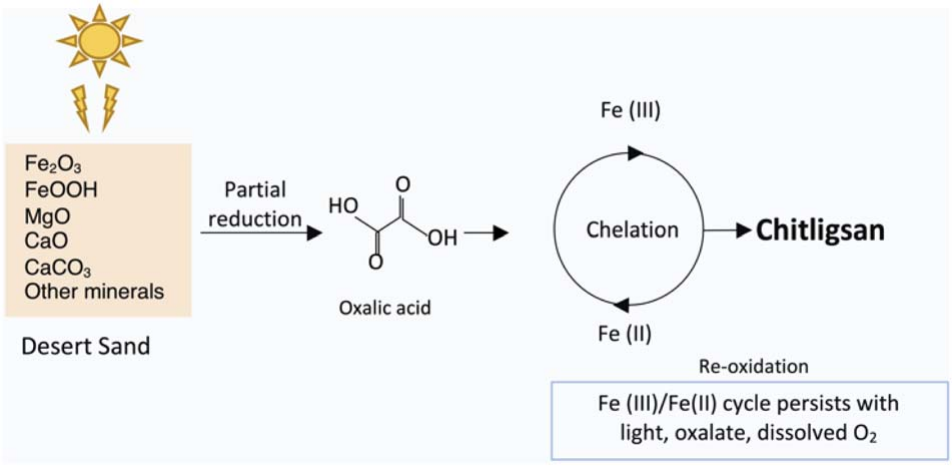
Proposed formation mechanism of Chitligsan. Desert sand minerals (Fe_2_O_3_, FeOOH) interact with oxalic acid and light, promoting Fe(iii)/Fe(ii) redox cycling through chelation and partial reduction under light and moisture, leading to the formation of the mineral composite Chitligsan.

The chemical formation of Chitligsan can be interpreted through the interactions among oxalic acid, chitin, and Fe-containing minerals. The schematic illustration (Fig. 5) summarizes this proposed pathway. Oxalic acid likely acts as a strong chelating and reducing agent, binding Fe^3+^ ions *via* its carboxyl groups and letting them partial photochemically reduce to Fe^2+^. This principle has already been demonstrated for oxalate-iron complexes in Sahara Desert dust systems.^14^ Ligand-assisted Fe(iii)/Fe(ii) transitions have also been documented in dust systems, where low-pH and ligand-rich environments enhance Fe-oxide dissolution and partial reduction.^51^

This photo- and chemically driven Fe(iii)/Fe(ii) redox cycle likely occurred during synthesis, with the resulting composite retaining surface-bound Fe species that may display limited redox reactivity under ambient conditions. The bioreactor synthesis of Chitligsan may reproduce similar redox and binding reactions, forming a mineral composite that could keep redox-active sites linked to its antibacterial effect. In our system, Fe–Ti oxide phases such as ilmenite (FeTiO_3_), identified by XRD, may participate in ligand-assisted Fe^3+^/Fe^2+^ cycling, stabilizing mixed-valence states during synthesis. According to Ivanyuk *et al.*, Fe–Ti mixed-valence oxides contain both Fe^2+^ and Fe^3+^ ions in octahedral coordination, enabling internal electron transfer between them.^52^ FTIR spectra showing metal-O and carboxylate vibrations, together with XRF evidence of Fe and Ca coordination, suggest strong metal–ligand interactions within the composite. These coordination features are consistent with natural Fe–ligand systems that can support redox transitions, which may indirectly contribute to the observed antibacterial behavior.^53^

### 3.2. Antibacterial activity of Chitligsan

The antibacterial activities of the Chitligsan-distilled water (Chitligsan-DI) solution and the Chitligsan-citric acid solution were analysed against one Gram-positive bacterium, *Staphylococcus aureus* ATCC 29213, and two Gram-negative bacteria, *Escherichia coli* ATCC 25922 and *Pseudomonas aeruginosa* ATCC 27853, using the agar well diffusion method.

**Fig. 6:**
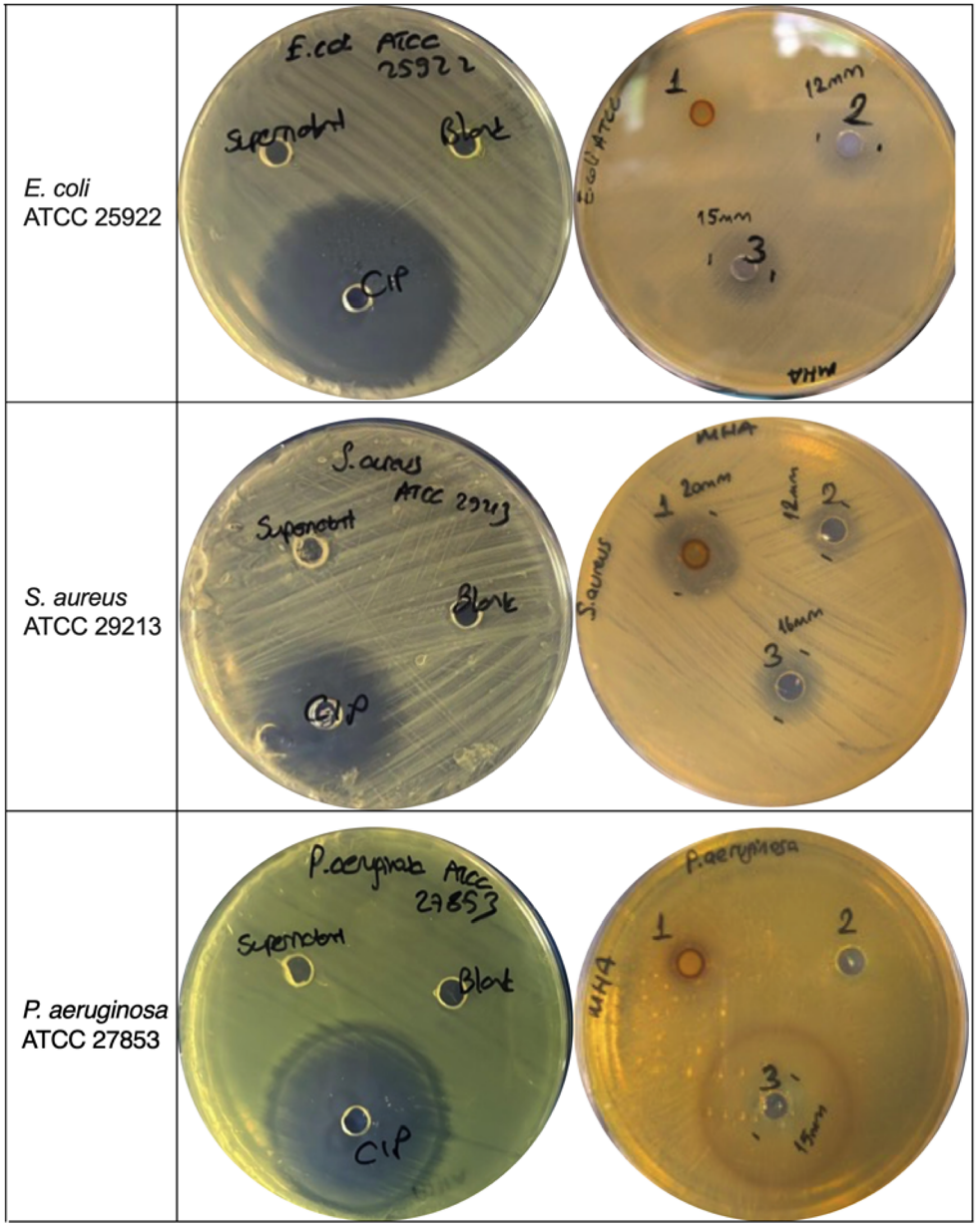
Antibacterial activities of Chitligsan samples, prepared with distilled water and 0.1 M citric acid, against *E. coli* (ATCC 25922), *S. aureus* (ATCC 29213), and *P. aeruginosa* (ATCC 27853). Supernatant: supernatant of Chitligsan suspension, blank: distilled water (DI), ciprofloxacin (CIP) (5 µg mL^−1^), 1: Chitligsan DI extract (1 mg mL^−1^), 2: 0.1 M citric acid (control), 3: Chitligsan 0.1 M citric acid extract (1 mg mL^−1^).

Regarding the results of antibacterial activity, Chitligsan-DI sample showed no antibacterial effect on *E. coli* and *P. aeruginosa*, but exhibited a notable growth suppression (inhibition zone diameter: 20 mm) against *S. aureus*. The Chitligsan-citric acid sample demonstrated inhibition zones of 16 mm for *S. aureus* and 15 mm for both *E. coli* and *P. aeruginosa* (Fig. 6). Citric acid alone was also tested as a control group, since it was used to obtain a homogeneous and diffusible Chitligsan suspension for agar-based antibacterial assay. Testing citric acid alone allowed us to assess whether it exhibited any intrinsic antibacterial activity. In contrast, the 0.1 M citric acid control group displayed lower activity against *E. coli* (inhibition zone diameter: 12 mm) and *S. aureus* (inhibition zone diameter: 12 mm), and no antibacterial activity against *P. aeruginosa*. These findings suggest that the antibacterial activity was clearly enhanced by Chitligsan samples (Table 2).

**Table 2:** Comparison of inhibition zone diameters (mm) of Chitligsan samples^a^

Bacteria	Chitligsan DI sample	Chitligsan 0.1 M citric acidic sample	0.1 M citric acid	Supernatant	Blank (DI)	CIP
*E. coli* ATCC 25922	0	15	12	0	0	50
*P. aeruginosa* ATCC 27853	0	15	0	0	0	45
*S. aureus* ATCC 29213	20	16	12	0	0	35

a Chitligsan-DI extract (1 mg mL^−1^), Chitligsan-citric acidic extract (1 mg mL^−1^), 0.1 M citric acid (pH 3.5); control, supernatant of the Chitligsan suspension, blank: distilled water, ciprofloxacin (CIP; 5 µg mL^−1^).

As a chelating agent, citric acid can enhance the solubility and stability of metal ions such as Fe^3+^, Zn^2+^, Mg^2+^ and Ag^+^ in aqueous media.^54–57^ Moreover, in soil science and environmental chemistry, this weak organic acid is frequently used to remove metal ions from mineral matrices, such as clays and oxides.^58^ The use of citric acid with metal oxide nanoparticles has been shown to lead to an increase in the number of binding sites and the hydrophilic properties of the particles, as well as enhanced biocompatibility, ROS generation, and antimicrobial efficiency of the products.^59–61^ It may also enhance reactivity by modifying particle surfaces, potentially increasing ROS generation.^61^ Similarly, in our study, citric acid may have boosted antibacterial activity by promoting metal ion release and contributing to surface modification, thereby enhancing interaction with bacterial cells.

As previously mentioned, Chitligsan-DI sample displayed inhibition activity only against a Gram-positive bacterium, but the Chitligsan-citric acid sample showed efficacy against all bacterial strains tested. The antibacterial effect of the citric acid sample may be due to a higher release of active compounds, whereas DI may have been insufficient to generate or extract specific metal oxide compounds. This disparity in antibacterial efficacy of both sample solution might be related to the differences in structural composition of Gram-negative and Gram-positive bacterial cell walls. Gram-negative bacteria have an outer membrane rich in lipopolysaccharides, which may resist poorly dispersed nanoparticles.^62^ However, citric acid-modified nanostructures are likely to have overcome this barrier more effectively. Thus, the improved antimicrobial activity of the citric acid extract appears to stem from both chemical interactions and enhanced nanoparticle functionality. The outer membrane may limit the adherence of ROS and metal-based oxides such as Fe_2_O_3_, CaO, and SiO_2_ to the membrane structure. Additionally, citric acid has been shown to effectively reduce nanoparticle aggregation by increasing surface charge.^63,64^ Therefore, Chitligsan-citric acid sample appear to enhance the suppression of bacterial growth, likely due to their effective access to the cell walls of Gram-negative bacteria. Indeed, the compounds released from Chitligsan, when extracted with citric acid, demonstrated efficient and diverse bioactivity in this study.

Despite limited current research into the bioactivity of desert sands, promising studies, such as that by Ouchari *et al.*, have shown that Moroccan desert sand possesses notable antibacterial activity against both Gram-positive and Gram-negative bacteria.^65^ Minimum inhibitory concentration (MIC) assays were also conducted; however, upon mixing with the culture medium, the extract induced precipitation and noticeable pH alterations, which hindered reliable UV measurements. This behaviour is common in chitosan-based systems and frequently interferes with accurate MIC determination. In broth media, chitosan tends to aggregate and form visible precipitates, and pH variations can further alter its solubility and apparent activity, making it difficult to determine the true MIC value.^66^ Minor differences in molecular weight or deacetylation degree can also affect solubility and MIC values, reducing the reproducibility of assays performed under near-neutral conditions.^67^ Detailed procedures and corresponding data are provided in the SI (Fig. S6–S11). Beyond the direct application of desert sands, recent studies have focused on isolating desert-derived microorganisms and evaluating the antimicrobial properties of their metabolites. For instance, multiple Actinobacteria strains isolated from China's Kubuqi Desert have been reported to produce antimicrobial compounds, effectively inhibiting the growth of *E. coli* and *S. aureus*.^68^ These findings strongly suggest that desert sand represents a promising, underexplored source of antibacterial substances. To further advance this understanding, future research should focus on the specific interactions between the mineral components of desert sand and bacterial membranes.

Additionally, to evaluate the effect of Chitligsan against antibiotic-resistant bacteria, methicillin-resistant *S. aureus* (MRSA ATCC 43300) was investigated; however, no discernible inhibitory activity was detected under the tested conditions. This outcome may be associated with the strain's strong oxidative stress tolerance and biofilm-forming capacity, which are known to reduce susceptibility to ROS-related antibacterial mechanisms, as reported in the literature.^69^ Representative images for MRSA assay are presented in the SI (Fig. S5).

**Fig. 7:**
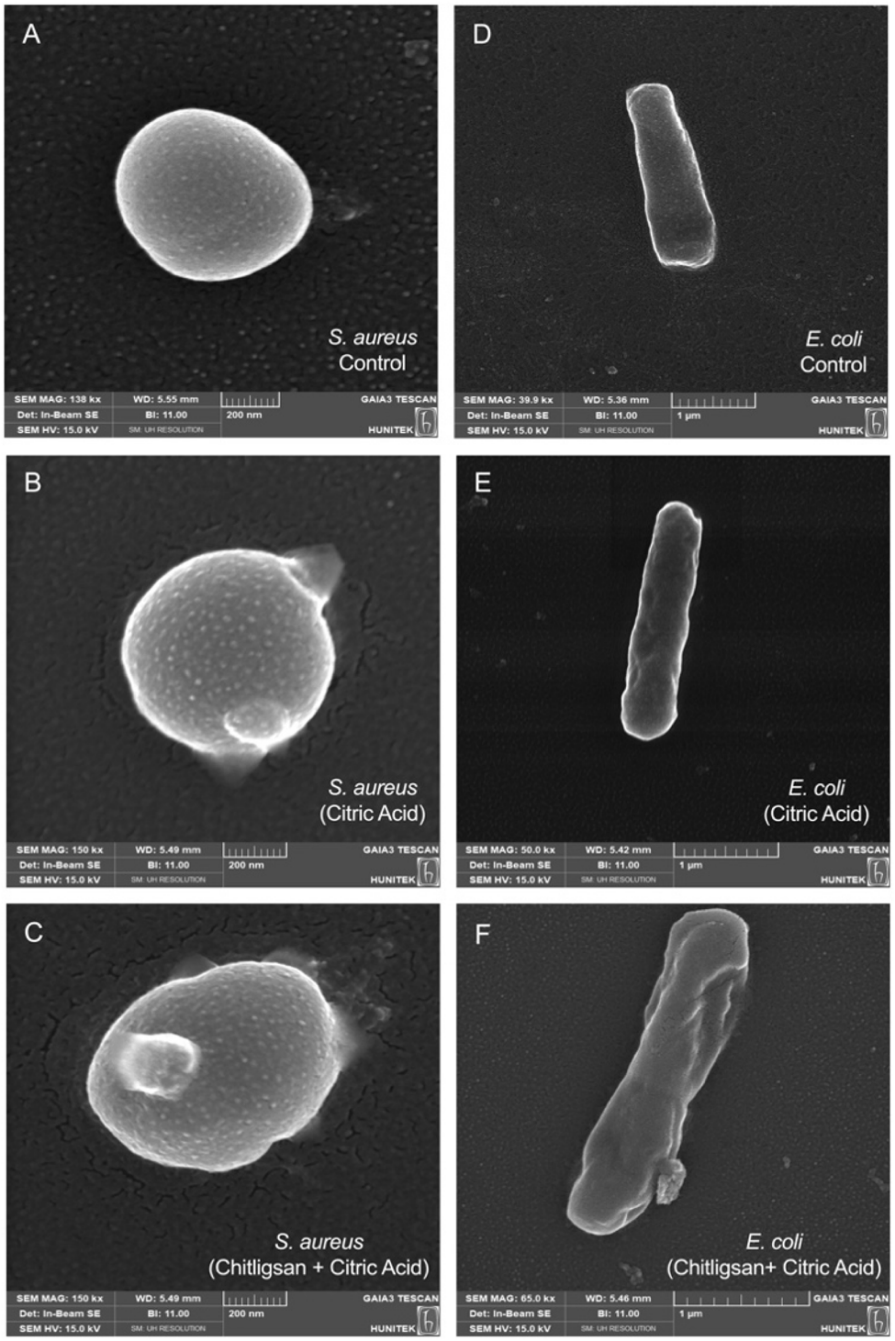
SEM micrographs illustrating the morphological changes of *S. aureus* (A–C) and *E. coli* (D–F) upon exposure to Chitligsan or citric acid. (A and D) Untreated control cells; (B and E) cells treated with 0.1 M citric acid; (C and F) cells treated with Chitligsan (1 mg mL^−1^) and 0.1 M citric acid.

In line with these observations, the present study also examined the antibacterial effects of Chitligsan and visualized its interaction with bacterial cells at the microscopic level. Scanning electron microscopy (SEM) was employed to visualize the morphological alterations in both Gram-positive (*S. aureus* ATCC 29213) and Gram-negative (*E. coli* ATCC 25922) bacteria after exposure to citric acid alone and to the Chitligsan composite dissolved in citric acid. In the control samples (Fig. 7A and D), both bacteria displayed smooth, intact, and turgid cell surfaces, characteristic of healthy cellular morphology. Cells treated solely with citric acid (Fig. 7B and E) exhibited mild surface roughness and localized deformation, suggesting partial stress on the cell envelope but not complete structural collapse. In contrast, exposure to the Chitligsan + citric acid (Fig. 7C and F) caused pronounced distortions of the cellular outline, including surface wrinkling, shrinkage, and membrane disruption, indicative of severe envelope damage and loss of cellular integrity.

These SEM observations are consistent with previous reports describing antimicrobial agents that compromise bacterial membranes or cell-wall stability. Hartmann *et al.* and Cushnie *et al.* reported similar topographical changes-surface blistering, contraction, and collapse-following exposure to reactive or metal-oxide-based materials,^70,71^ while Yaacob *et al.* demonstrated comparable envelope disintegration under acidic stress.^72^ Likewise, Hisada *et al.* confirmed that such morphological deformities (swelling, bulging, and lysis) correspond to lethal damage in susceptible bacteria when the cell envelope integrity is disrupted.^73^ Therefore, the severe deformation observed in the Chitligsan-treated samples supports the hypothesis that this hybrid organic–inorganic polymer exerts a bactericidal effect primarily through cell-envelope destabilization, possibly enhanced by surface charge interactions and metal-oxide components that intensify oxidative or electrostatic stress on the bacterial membrane.

### 3.3. Assessment of Chitligsan's *in vitro* cytocompatibility

**Fig. 8:**
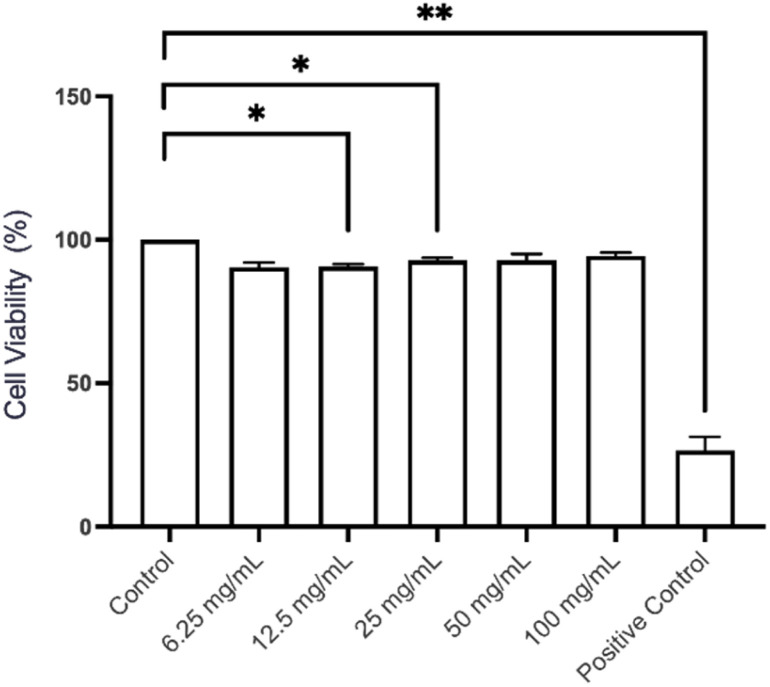
MTT assay results for L929 cells after 24 h of exposure to serial dilutions (50 mg mL^−1^, 25 mg mL^−1^, 12.5 mg mL^−1^, 6.25 mg mL^−1^) of Chitligsan stock solution (100 mg mL ^−1^). *N* = 3, *p < 0.05; **p < 0.01. Control: untreated cells. Positive control (cytotoxic agent): 2.5% DMSO.

To assess the potential of Chitligsan as an antimicrobial agent, its cytocompatibility was evaluated using an MTT assay on L929 mouse fibroblast cells. The L929 cells were incubated for 24 h with serial dilutions of a 100 mg per mL Chitligsan stock solution (50 mg mL^−1^, 25 mg mL^−1^, 12.5 mg mL^−1^, 6.25 mg mL^−1^) prepared in RPMI-1640 medium. The results revealed a generally well-tolerated profile for Chitligsan, which exhibited high cytocompatibility (Fig. 8).

While a statistically significant decrease in cell viability (*p* < 0.05) was observed across all concentrations, the most pronounced reduction only reached 90.5% and was observed in the group treated with 6.25 mg per mL Chitligsan: RPMI-1640 medium. Importantly, no dose-dependent cytotoxic trend was detected, and cell viability remained well above the commonly accepted cytotoxicity threshold of 70%. These findings indicate that Chitligsan maintains high cellular viability even at elevated concentrations, supporting its cytocompatibility and suitability for further biomedical and antimicrobial applications.

### 3.4. Evaluation of *in vitro* cell migration and cytocompatibility

**Fig. 9:**
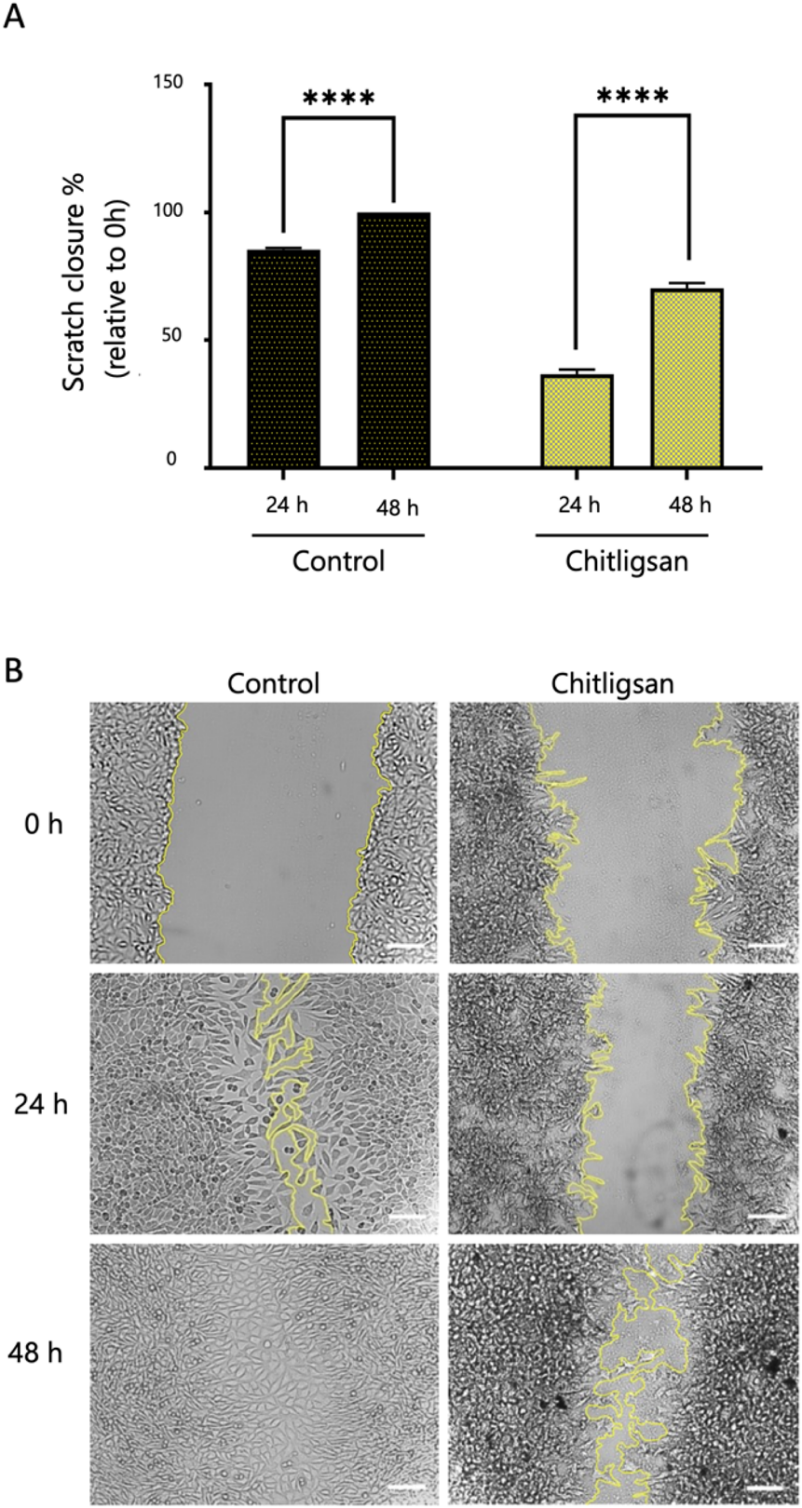
Scratch assay results for L929 cells after 48 h of exposure to Chitligsan (100 mg mL^−1^). Quantification of scratch closure at 24 h and 48 h relative to 0 h in control and Chitligsan-treated groups. The scale bar in the images represents 100 µm. Control: untreated cells. Quantitative analysis of scratch closure (%) at 24 h and 48 h relative to 0 h. (A) Quantification of scratch closure at 24 h and 48 h relative to 0 h, (B) Representative phase-contrast images of scratch wounds at 0 h, 24 h, and 48 h.

A scratch assay was employed to assess cell migration as an indicator of cytocompatibility following Chitligsan treatment (Fig. 9). The control group exhibited approximately 85% scratch closure at 24 hours, reaching confluency (100%) by 48 hours. Cells treated with Chitligsan solution (without media) demonstrated a reduced migration rate, achieving approximately 36% closure at 24 hours and around 70% closure at 48 hours (*p* < 0.0001) compared to the control group at both time points.

The delayed rate observed in the presence of the material is attributed primarily to a modulation of cell migration and/or proliferation rather than a loss of cell viability. Unlike conventional scratch assay conditions in which test materials are supplemented into complete culture medium, the material was applied as a standalone solution to investigate its direct interaction with cells in the absence of exogenous growth factors. Under these nutrient-limited conditions, cell proliferation is expected to be reduced, which contributes to the slower closure kinetics. Nevertheless, the sustained cell viability demonstrates that the material provides a biocompatible microenvironment capable of supporting cell survival.

In addition, the nanostructured mineral–biopolymer composition of the material may further influence cellular behavior by modulating cell–substrate interactions, cytoskeletal organization, and focal adhesion dynamics. The presence of nanoflower-like architectures and potential ion-mediated signaling effects may result in a cytostatic but non-cytotoxic response, characterized by reduced migration rates without triggering apoptotic or necrotic pathways.^74^

However, in one of the recent study, Chitligsan-based formulations have also been evaluated in *in vivo* wound models, where beneficial effects on wound closure and tissue regeneration were reported.^15^ In that study, the effects of Chitligsan on wound surface area were compared with those of tetramycin, an antibiotic known for its wound-healing effect, at different time points. Chitligsan-based gel and spray formulations significantly enhanced wound closure, epithelial regeneration, and collagen formation.^15^

The observed differences between the present *in vitro* findings and previously reported *in vivo* outcomes may stem from fundamental differences between *in vitro* and *in vivo* experimental systems. *In vivo* systems are influenced by a complex interplay of immune responses, genetic factors, metabolism, and cell-to-cell interactions. Therefore, *in vitro* data may not always accurately reflect the actual *in vivo* conditions.^52^

Overall, the *in vitro* scratch assay results indicate that Chitligsan is biocompatible and capable of modulating cell migration kinetics, rather than exerting a detrimental effect on cell viability, highlighting its potential suitability for advanced biomedical and regenerative applications.

These findings support Chitligsan as a biocompatible, non-cytotoxic, and cost-effective natural alternative to conventional metal-based systems widely employed in biotechnology. The synthesis of Chitligsan follows a biomimetic pathway similar to natural geochemical processes. Similar sunlight, moisture and fungal-origin oxalic acid driven transformations of metal oxides have been reported in desert dust systems.^14^ Minerals such as Fe_2_O_3_, SiO_2_, and CaO have been reported by Journet *et al.* as major components of dust influencing iron solubility.^51^ Although detailed biodegradability were not performed in this study, based on the inorganic compositions identified in Chitligsan, we propose that similar mineral forms may also be present, suggesting comparable geochemical stability under ambient conditions. This compositional persistence suggests that the compound is chemically stable and environmentally compatible, though not readily biodegradable, and its environmental safety should be interpreted within this context.

## 4 Conclusion

This study focused on physicochemical characteristics, antibacterial efficacy, and cytocompatibility of Chitligsan, which is naturally derived from Sahara Desert sand can be a promising alternative material for biomedical applications.

Our analyses revealed that Chitligsan particles possess diverse nanostructures and are rich in various metal oxides and elements, including Fe, Mg, Na, Al, Si, Ca, and Ti. These findings were consistently supported by a range of complementary analytical techniques, confirming the presence of key minerals such as Calcite, Hydrotalcite, Clinopyroxene and Ilmenite, which likely contribute to its unique properties. The mineral composition of Chitligsan gives it a reactive surface and a clear pH-dependent colloidal behaviour; under alkaline conditions, the particles tend to associate and aggregate, which may influence their stability and bioavailability.

Chitligsan extracted with citric acid demonstrated significant antibacterial activity, which can be attributed to enhanced ionic interactions in an acidic environment. This antibacterial activity likely stems from the diverse metal oxides within Chitligsan, as their antimicrobial effects are well-documented. In addition, the material displayed organic signatures characteristic of chitosan-like biopolymers, including nitrogen-containing heterocycles such as pyrroles, pyrazines, and pyridines, suggesting that part of its complex polymeric framework may originate from residual biopolymeric components. Furthermore, the material exhibited notable biocompatibility with fibroblast cells, indicating a high level of cell viability. While initial *in vitro* observations indicated a slower rate of cell migration, cells remained viable and morphologically intact throughout the experiment, demonstrating the cytocompatibility of Chitligsan.

As a renewable, abundant, and cost-effective natural source of metal oxide nanostructures, Chitligsan offers a combination of antibacterial activity and biocompatibility. Its unique attributes suggest its suitability for diverse medical or veterinarian applications, whether as a standalone material or integrated into various delivery systems like gels, or sprays.

## Author contributions

Gamze Ertunc: investigation, methodology, formal analysis, validation, data curation, writing – original draft. Ebru Yilmaz: investigation, data curation, formal analysis, visualization, writing – original draft. Dilan Celebi-Birand: investigation, formal analysis, visualization, writing – review & editing. Busra Kılıc: investigation, methodology, visualization, formal analysis, writing – review & editing. Şeyma Nigiz: investigation, methodology, visualization, writing – review & editing. Evren Çubukçu: investigation, methodology, visualization, writing – review & editing. Ceren Özkul: methodology, writing – review & editing. Halil Murat Aydın: methodology, writing – review & editing. Memed Duman: conceptualization, funding acquisition, writing – review & editing, resources, supervision, project administration.

## Conflicts of interest

There are no conflicts to declare.

## Supplementary Material

RA-016-D5RA04969E-s001

RA-016-D5RA04969E-s002

## Data Availability

Data are available upon request from the authors. Supplementary information (SI) is available. See DOI: https://doi.org/10.1039/d5ra04969e.
