# Supplementary material for: Sahara Desert sand “Chitligsan”: characterisation and assessment of antibacterial activity and cytotoxicity
Source: RSC Adv. 2026 Jan 15;16(4):3395–408. doi: 10.1039/d5ra04969e (PMC12805422; doi:10.1039/d5ra04969e)
Supplement: RA-016-D5RA04969E-s002 [file RA-016-D5RA04969E-s002.pdf]

Supplementary Information (SI) for RSC Advances.
This journal is © The Royal Society of Chemistry 2026

Supporting Information

Sahara Desert Sand "Chitligsan": Characterisation and Assessment of Antibacterial Activity and Cytotoxicity

Gamze Ertunc [a], Ebru Yilmaz [a], Dilan Celebi-Birand [a,f], Busra Kılıc [b], Seyma Nigiz [c], Evren Cubukcu [d], Ceren Özkul[c], Halil Murat Aydin [e], Memed Duman [a]

[a]Hacettepe University, Institute of Science, Nanotechnology and Nanomedicine Division, Ankara, Türkiye.

[b]Molecular Biology Section, Department of Biology, Faculty of Science, Hacettepe University, Beytepe, Ankara, Türkiye.

[c]Hacettepe University Faculty of Pharmacy, Department of Pharmaceutical Microbiology, Ankara, Türkiye.

[d]Hacettepe University, Faculty of Engineering, Department of Geological Engineering, Beytepe Campus, Ankara, Türkiye.

[e]Bioengineering Division and Centre for Bioengineering, Institute of Science, Hacettepe University, Beytepe, Ankara, Türkiye.

[f]Department of Molecular Biology and Genetics, Bilkent University, Ankara, Türkiye.

Results and Discussion

Zeta Potential & DLS

A

Results

			Mean (mV)	Area (%)	St Dev (mV)
Zeta Potential (mV):	-0,409	Peak 1:	-0,409	100,0	4,30
Zeta Deviation (mV):	4,30	Peak 2:	0,00	0,0	0,00
Conductivity (mS/cm):	1,04	Peak 3:	0,00	0,0	0,00
Result quality :	See result quality report				

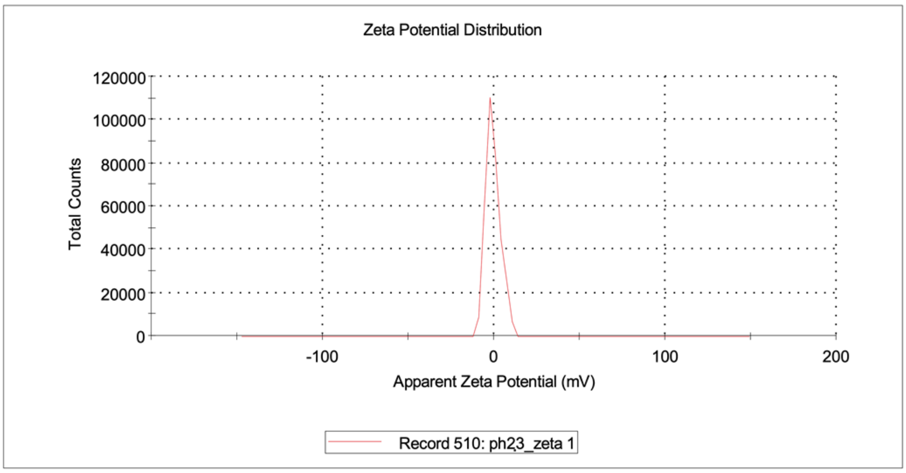

B

Results

			Size (d.nm):	% Intensity:	St Dev (d.n...
Z-Average (d.nm):	1495	Peak 1:	369,7	94,3	49,89
PdI:	0,835	Peak 2:	51,19	5,7	5,144
Intercept:	0,886	Peak 3:	0,000	0,0	0,000
Result quality :	Refer to quality report				

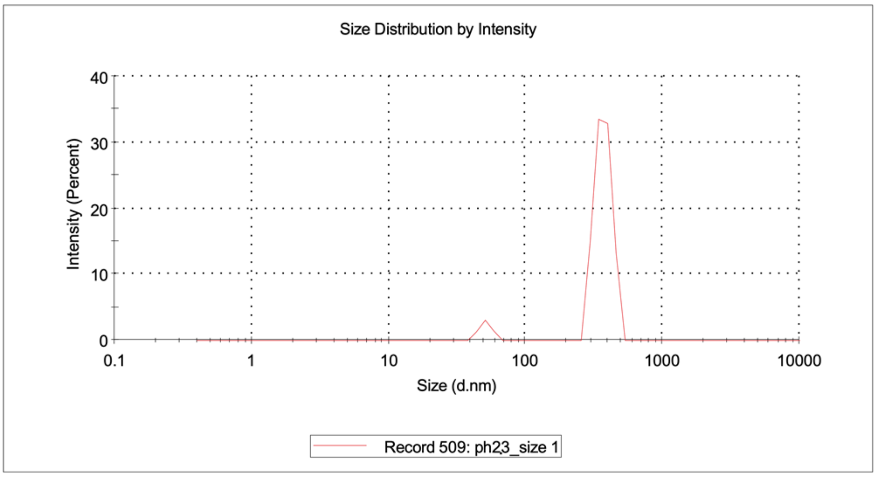

Fig. S1. The zeta potential (A) and particle size (B) and analysis of Chitligsan at pH 2.3

A

Results

			Mean (mV)	Area (%)	St Dev (mV)
Zeta Potential (mV):	-1,74	Peak 1:	-1,74	100,0	3,47
Zeta Deviation (mV):	3,47	Peak 2:	0,00	0,0	0,00
Conductivity (mS/cm):	0,298	Peak 3:	0,00	0,0	0,00
Result quality :	See result quality report				

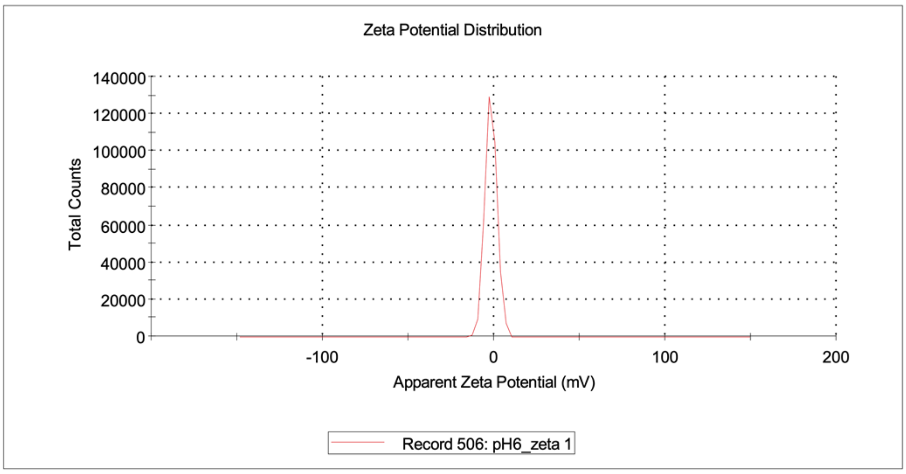

B

Results

			Size (d.nm):	% Intensity:	St Dev (d.n...
Z-Average (d.nm):	659,7	Peak 1:	362,3	81,2	70,15
PdI:	0,594	Peak 2:	103,8	18,8	16,53
Intercept:	0,902	Peak 3:	0,000	0,0	0,000
Result quality :	Refer to quality report				

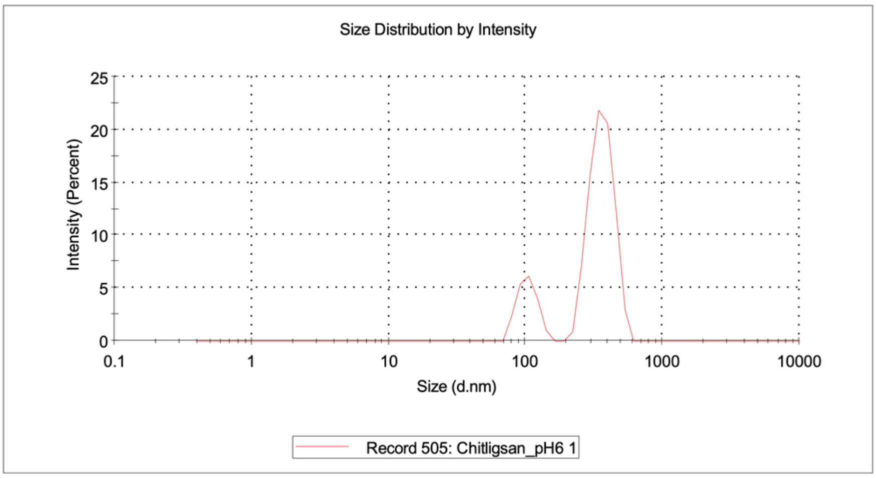

Fig. S2. The zeta potential (A) and particle size (B) and analysis of Chitligsan at pH 6.

A

Results

			Mean (mV)	Area (%)	St Dev (mV)
Zeta Potential (mV):	-6,29	Peak 1:	-5,76	92,6	11,0
Zeta Deviation (mV):	27,3	Peak 2:	24,1	7,1	3,74
Conductivity (mS/cm):	3,64	Peak 3:	39,8	0,4	2,40

Result quality : See result quality report

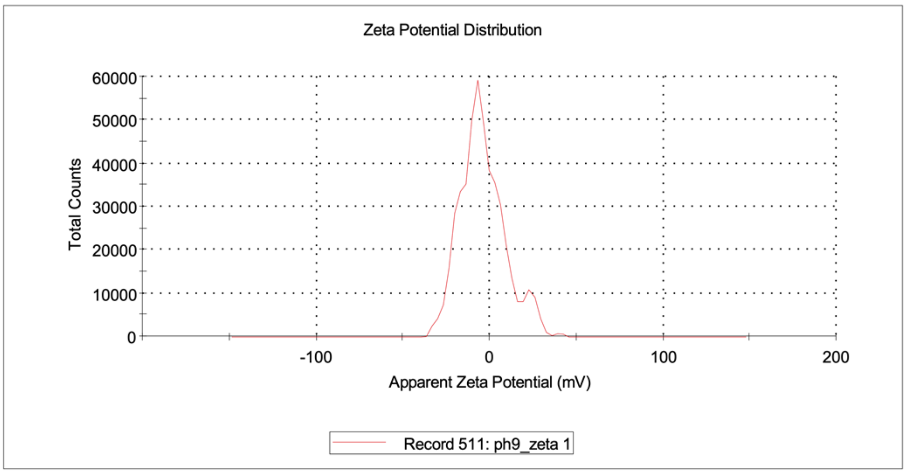

B

Results

			Size (d.nm):	% Intensity:	St Dev (d.n...
Z-Average (d.nm):	505,1	Peak 1:	645,4	71,9	212,4
PdI:	0,620	Peak 2:	195,5	25,6	48,86
Intercept:	0,919	Peak 3:	5480	2,5	233,0

Result quality : Refer to quality report

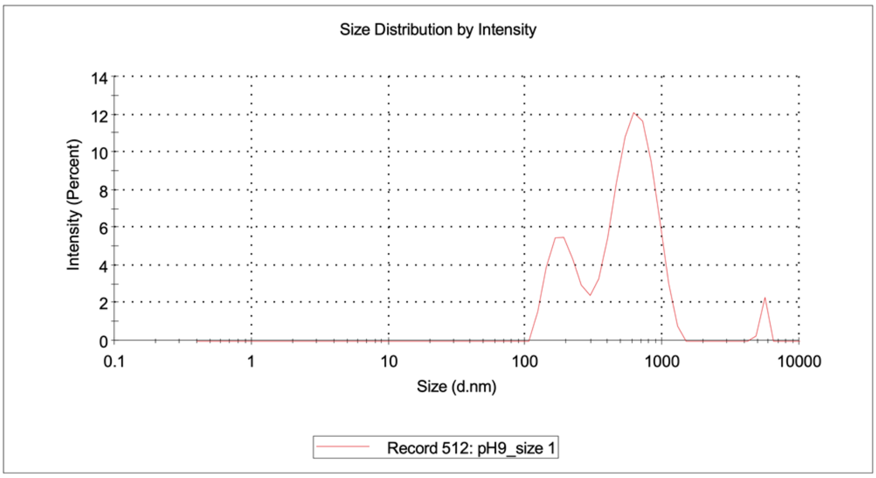

Fig. S3. The zeta potential (A) and particle size (B) and analysis of Chitligsan at pH 9.

A

Results

			Mean (mV)	Area (%)	St Dev (mV)
Zeta Potential (mV):	4,33	Peak 1:	4,33	100,0	4,73
Zeta Deviation (mV):	4,73	Peak 2:	0,00	0,0	0,00
Conductivity (mS/cm):	0,268	Peak 3:	0,00	0,0	0,00
Result quality :	Good				

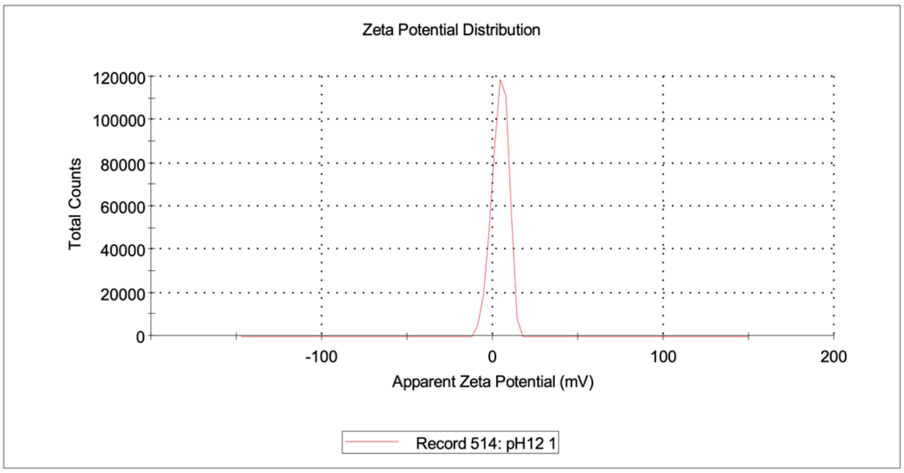

B

Results

			Size (d.nm):	% Intensity:	St Dev (d.n...
Z-Average (d.nm):	1186	Peak 1:	1007	94,4	248,4
PdI:	0,535	Peak 2:	147,6	5,6	22,66
Intercept:	0,895	Peak 3:	0,000	0,0	0,000
Result quality :	Refer to quality report				

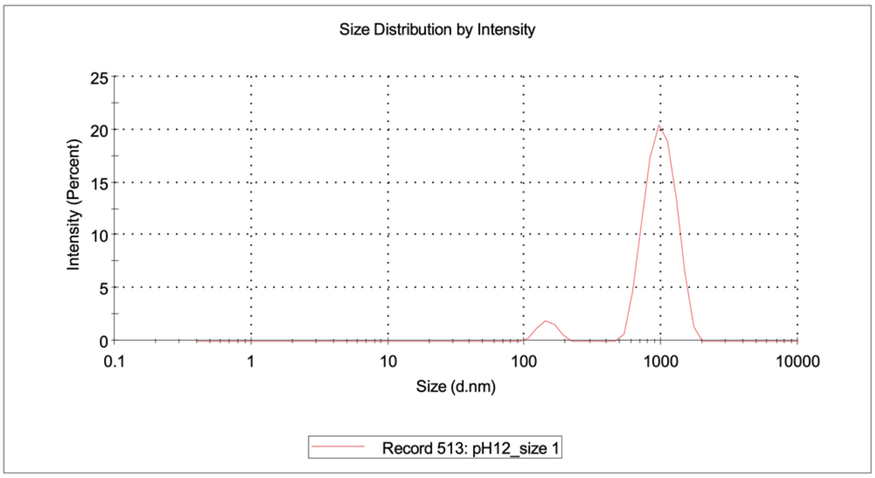

Fig. S4. The zeta potential (A) and particle size (B) and analysis of Chitligsan at pH 12.

Antibacterial Activity

Determination of antimicrobial activity against methicillin-resistant *Staphylococcus aureus* ATCC 43300 (MRSA ATCC 43300).

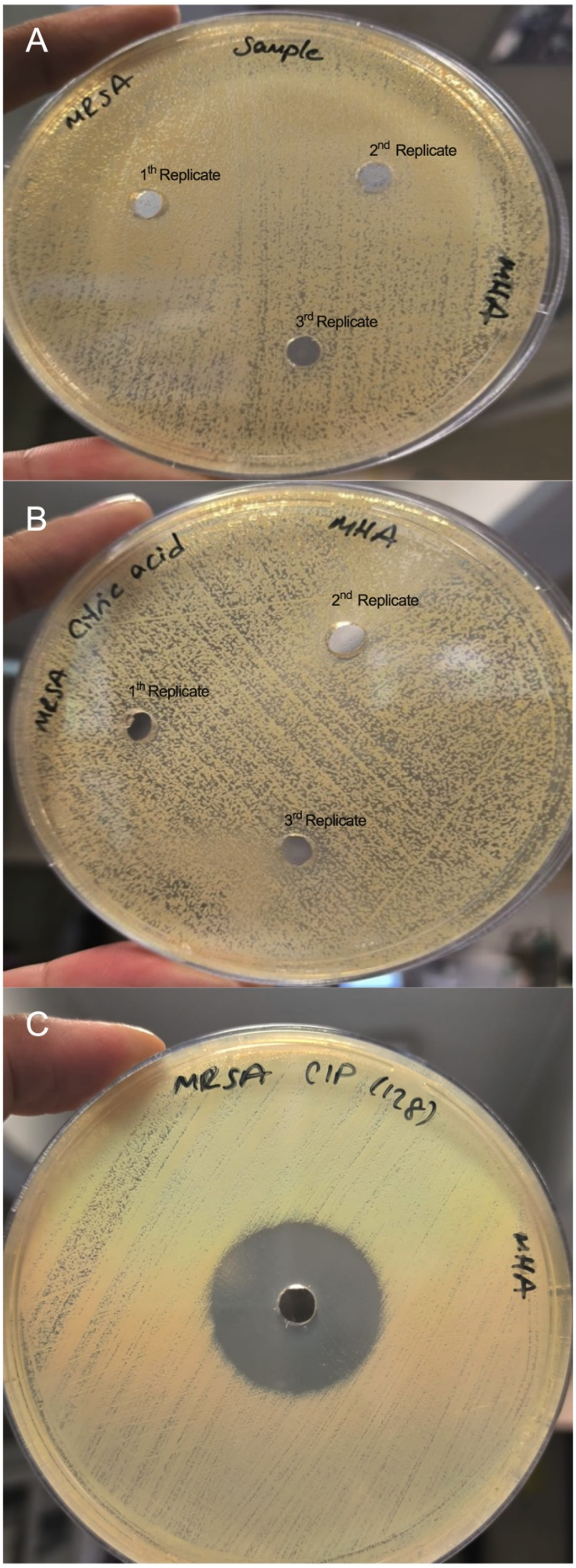

Fig. S5. Antibacterial evaluation of Chitligsan extracts against methicillin-resistant *Staphylococcus aureus* (MRSA ATCC 43300) using the agar well diffusion method. (A) Chitligsan–0.1 M citric acid extract, (B) 0.1 M citric acid, and (C) Ciprofloxacin as a positive control. All assays were performed in three independent replicates.

Determination of antimicrobial activity by broth microdilution method

In *vitro* antimicrobial activity assays were carried out by broth microdilution method according to the Clinical and Laboratory Standards Institute (CLSI) guidelines against a panel of reference bacterial strains including *E. coli* ATCC 25922, *S. aureus* ATCC 29213, *P. aeruginosa* ATCC [1]. In addition, methicillin-resistant *S. aureus* (MRSA) ATCC 43300 was also included to evaluate the activity against a resistant strain. Chitligsan-0.1 M citric acid extract and 0.1 M citric acid were prepared in 96-well microplates using two-fold serial dilutions to obtain a range of test concentrations. Ciprofloxacin was used as control drugs for tested bacteria. Bacterial suspensions were prepared in sterile PBS at a density of 0.5 McFarland turbidity standard from fresh cultures of standard bacterial strains on Tryptic Soy Agar (TSA, Merck). The suspensions were subsequently diluted in MHB and added to each well to give a final cell density of 5×10^5 cfu/mL. Sterility control and growth control were included in each assay. MICs were read after incubation at 37°C for 24 h.

Reference

[1] Clinical and Laboratory Standards Institute (CLSI). Methods for dilution antimicrobial susceptibility tests for bacteria that grow aerobically, 10th ed Approved standard M07-A10, Wayne, PA, USA., (2015).

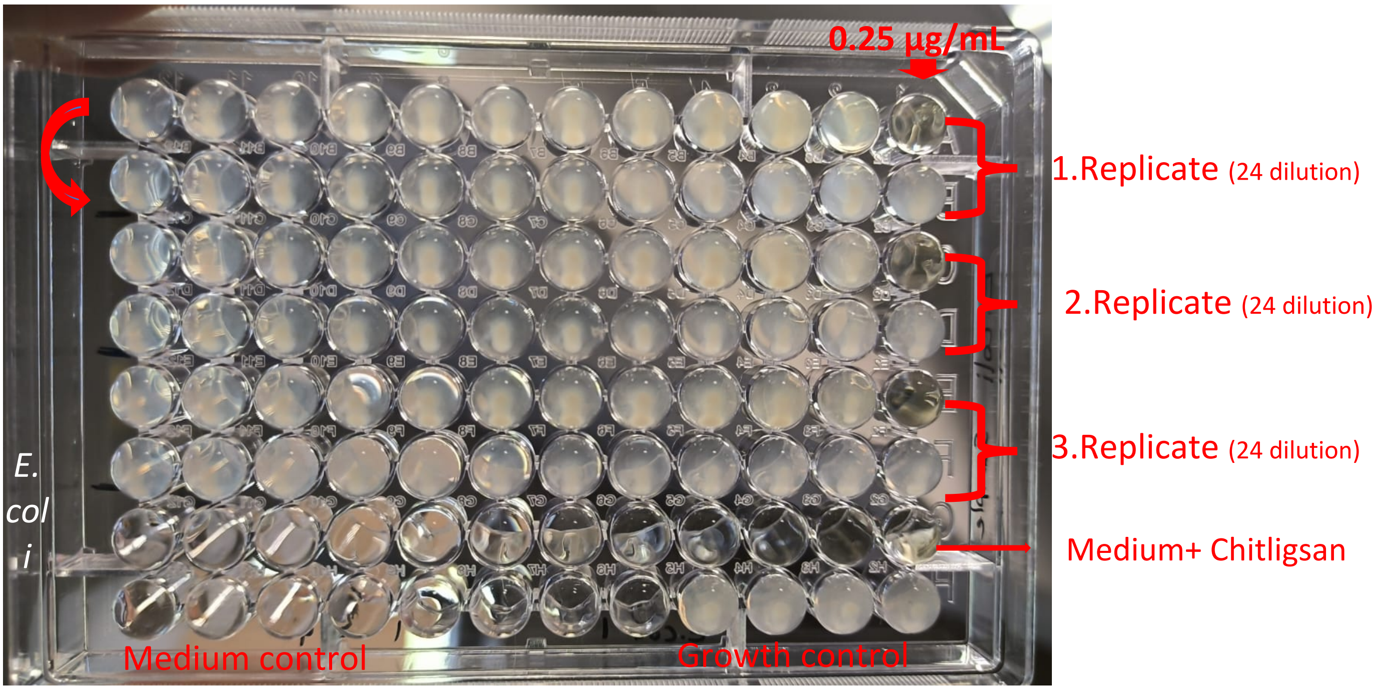

Fig. S6. Broth microdilution assay of *Escherichia coli* treated with Chitligsan–citric acid extract. The extract was serially diluted (two-fold, 24 steps) and evaluated in triplicate.

Reliable readings could be obtained only up to concentrations that remained stable in the broth, as precipitation limited further assessment.

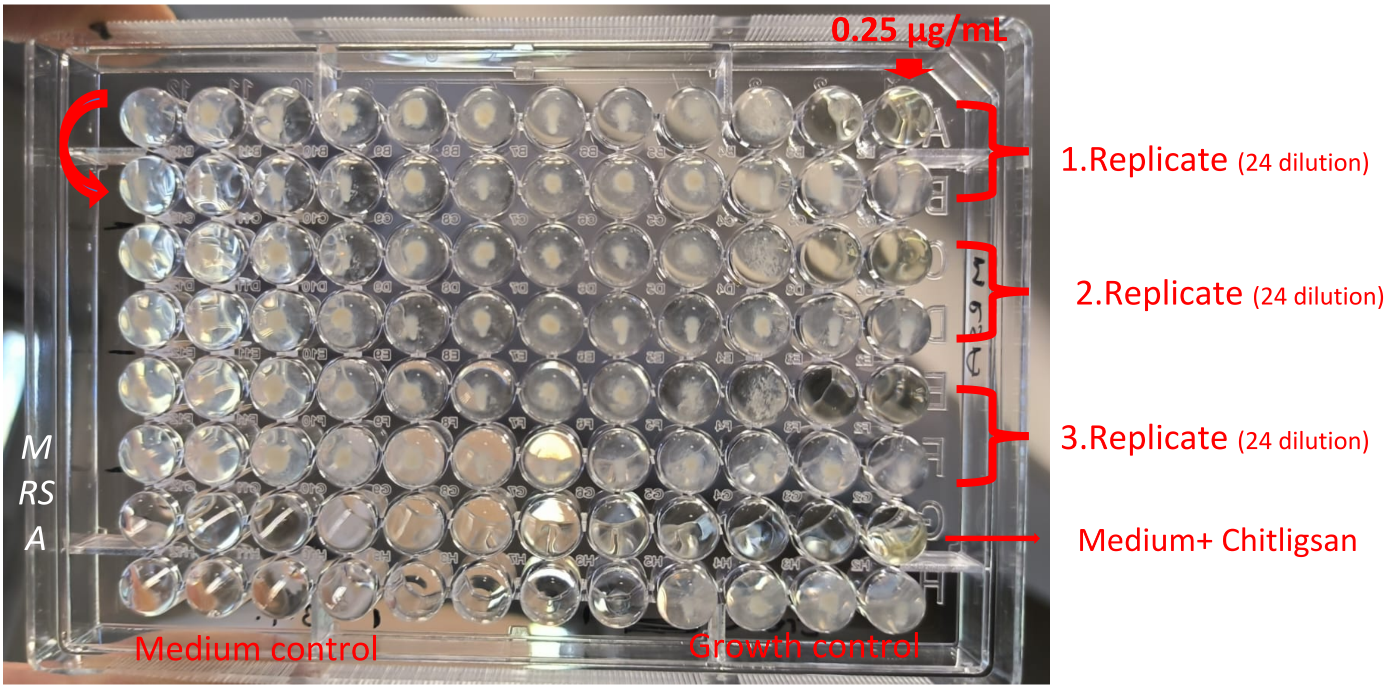

Fig. S7. Broth microdilution assay of Methicillin-resistant *Staphylococcus aureus* (MRSA) treated with Chitligsan–citric acid extract. The extract was serially diluted (two-fold, 24 steps) and evaluated in triplicate.

Reliable readings could be obtained only up to concentrations that remained stable in the broth, as precipitation limited further assessment.

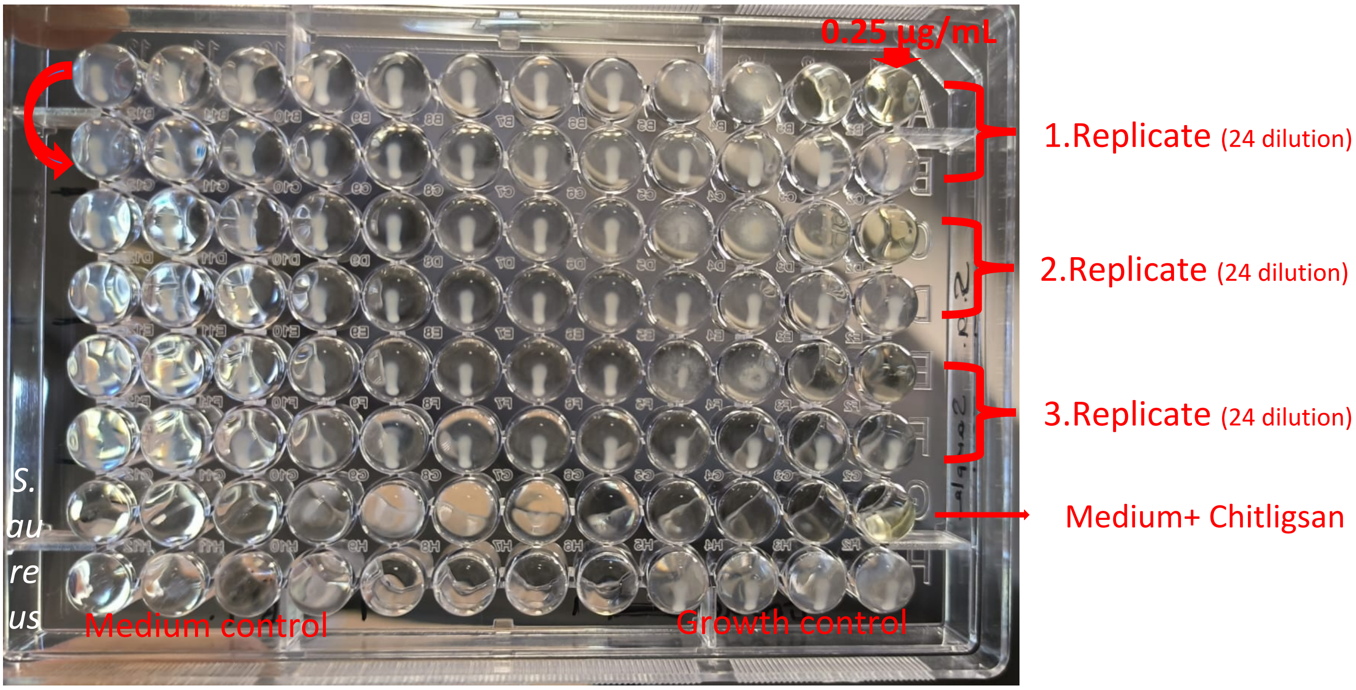

Fig. S8. Broth microdilution assay *Staphylococcus aureus* treated with Chitligsan–citric acid extract. The extract was serially diluted (two-fold, 24 steps) and evaluated in triplicate. Reliable readings could be obtained only up to concentrations that remained stable in the broth, as precipitation limited further assessment.

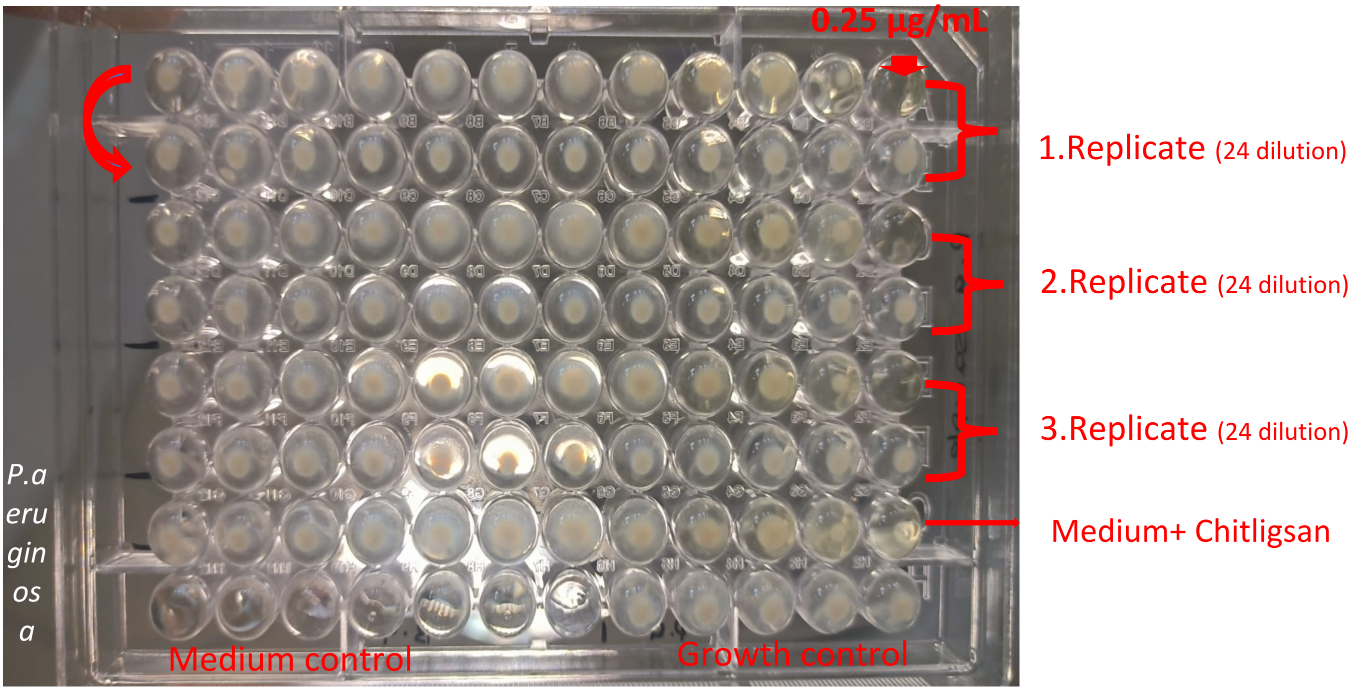

Fig. S9. Broth microdilution assay *Pseudomonas aeruginosa* treated with Chitligsan–citric acid extract. The extract was serially diluted (two-fold, 24 steps) and evaluated in triplicate. Reliable readings could be obtained only up to concentrations that remained stable in the broth, as precipitation limited further assessment.

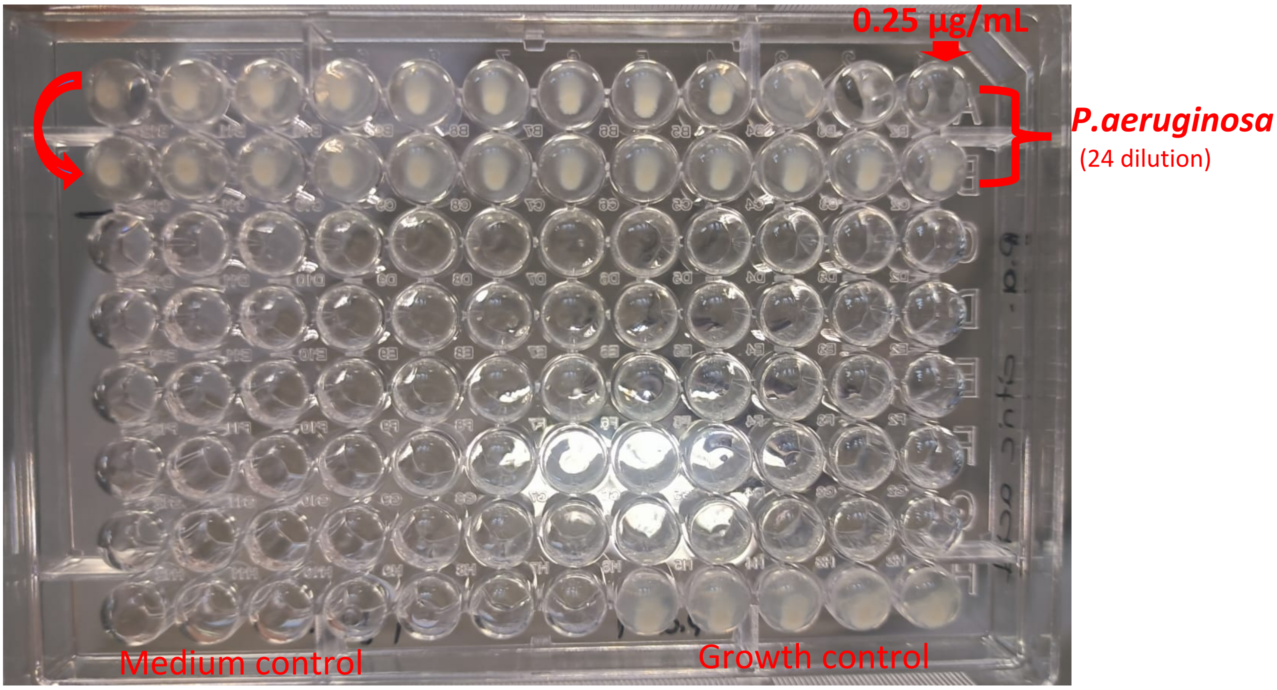

Fig. S10. Broth microdilution assay *Pseudomonas aeruginosa* treated with 0.1 M citric acid. The extract was serially diluted (two-fold, 24 steps) and evaluated in triplicate.

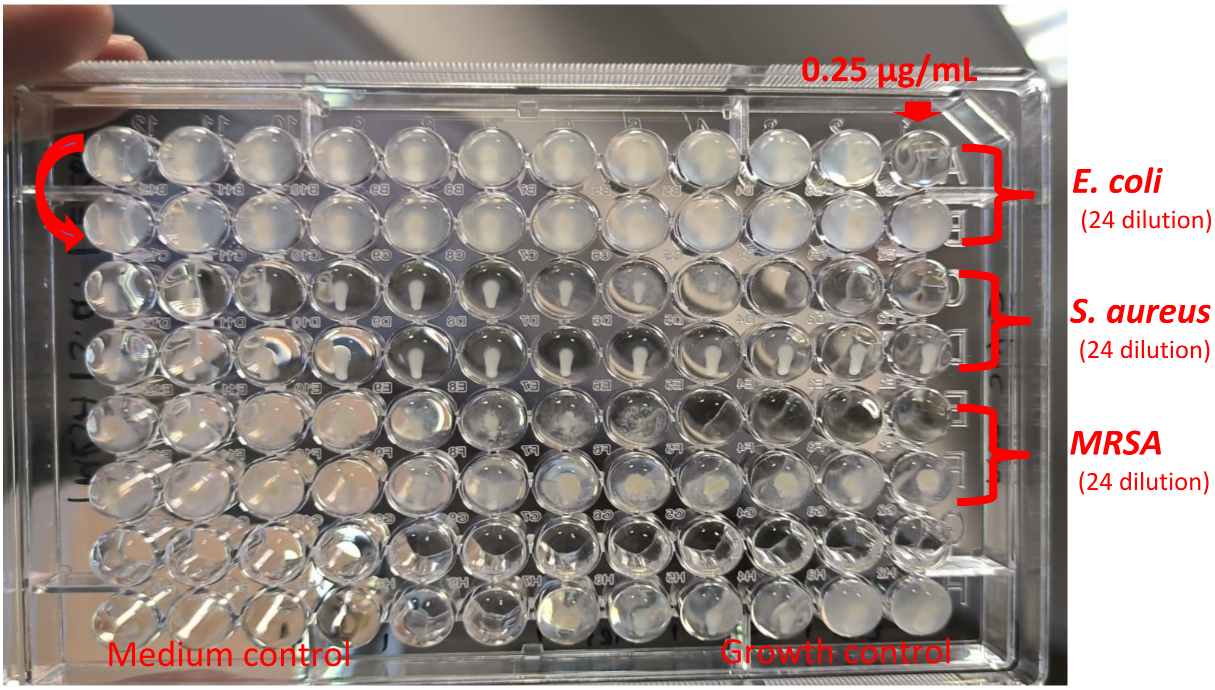

Fig. S11. Broth microdilution assay *Escherichia coli, Staphylococcus aureus*, *and* Methicillin-resistant *Staphylococcus aureus* (MRSA) *aeruginosa* treated with 0.1 M citric acid. The extract was serially diluted (two-fold, 24 steps) and evaluated in triplicate.

Table S1. Minimum inhibitory concentrations (MIC) of Chitligsan–citric acid extract and 0.1 M citric acid, determined by the broth microdilution assay.

	E. coli ATCC 25922	*S. aureus* ATCC 29213	MRSA ATCC 43300	*P. aeruginosa* ATCC 27853
Chitligsan-citric acid extract (Initial concentration 0.25 μg/mL)	0.25 μg/mL	0.125 μg/mL	0.125 μg/mL	0.25 μg/mL
0.1 M Citric acid (Solvent control)	0.1 M	0.025 M	0.01 M	0.05 M

* Reliable readings could be obtained only up to concentrations that remained stable in the broth, as precipitation limited further assessment.
